# Mitochondria at the intersections of RNA modifications and metabolism reprogramming implications in cell death, tumor microenvironment, and immunotherapy

**DOI:** 10.1186/s13045-025-01762-7

**Published:** 2025-12-10

**Authors:** Jiaxun Zhang, Haoxuan Zhang, Leifeng Chen, Yuze Wu, Jiaming Xie, Yannan Yang, Aaron Chen, Akezhouli Shahatiaili, Shan Muhammad, Wenhui Yang, Yingli Sun, Yibo Gao

**Affiliations:** 1 Department of Thoracic Surgery, National Cancer Center/National Clinical Research Center for Cancer/Cancer Hospital, Chinese Academy of Medical Sciences and Peking Union Medical College, Beijing, 100021 China; 2 Department of Oncology, The Second Affiliated Hospital of Nanchang University, Nanchang, 330006 Jiangxi China; 3 Cancer Center, Renmin Hospital of Wuhan University, Wuhan, 430060 China; 4 Department of Thoracic Surgery, National Cancer Center/National Clinical Research Center for Cancer/Cancer Hospital & Shenzhen Hospital, Chinese Academy of Medical Sciences and Peking Union Medical College, Shenzhen, 518116 China; 5 University of Southern California, Los Angeles, CA USA; 6 Department of Gastroenterology, Shanxi Province Cancer Hospital/Shanxi Hospital Affiliated to Cancer Hospital, Chineses Academy of Medical Sciences/Cancer Hospital Affiliated to Shanxi Medical University, Taiyuan, 030013 China; 7 Central Laboratory & Shenzhen Key Laboratory of Epigenetics and Precision Medicine for Cancers, National Cancer Center/National Clinical Research Center for Cancer/Cancer Hospital & Shenzhen Hospital, Chinese Academy of Medical Sciences and Peking Union Medical College, Shenzhen, 518116 China; 8 Laboratory of Translational Medicine, National Cancer Center/National Clinical Research Center for Cancer/Cancer Hospital, Chinese Academy of Medical Sciences and Peking Union Medical College, Beijing, 100021 China; 9 State Key Laboratory of Molecular Oncology, National Cancer Center/National Clinical Research Center for Cancer/Cancer Hospital, Chinese Academy of Medical Sciences and Peking Union Medical College, Beijing, 100021 China

**Keywords:** RNA modifications, Mitochondria, Glucose metabolism, Regulated cell death, Mitochondrial dynamics, Cancer, Tumor microenvironment, Drug resistance, Aging-related disease

## Abstract

Mitochondria, the powerhouse of the cell, orchestrate a plethora of critical functions, including energy production, metabolic regulation, programmed cell death, and signal transduction. Their pivotal role in the pathogenesis of numerous diseases underscores their significance. Among the various regulatory mechanisms, RNA modifications emerge as a dominant posttranscriptional modulator of gene expression, increasingly recognized for their profound impact on mitochondrial functions. Groundbreaking discoveries have unveiled compelling links between RNA modifications and oxidative phosphorylation, regulated cell death—particularly cuproptosis—and antitumor immunity, underscoring RNA modifications’ vital role and untapped potential in mitochondrial biology, cancers and aging-related diseases. In this Review, we comprehensively catalog the primary RNA modifications modifiers and their small-molecule inhibitors that influence mitochondrial functions. We explore the latest research delineating RNA modifications’ involvement in mitochondria-related glucose metabolism, regulated cell death, and mitochondrial dynamics, presenting an intricate regulatory network. Furthermore, we investigate the intriguing intersection of RNA modifications and mitochondria-related antitumor immunity, highlighting prospective therapeutic targets to enhance immunotherapy outcomes. This review not only accentuates the critical importance of RNA modifications in mitochondrial function but also paves the way for novel therapeutic strategies in disease treatment.

## Background

Mitochondria function as versatile centers that are crucial for energy production, redox balance, biosynthetic capacity, cellular metabolism, programmed cell death, signal transduction, and intercellular organelle communication [1–3]. Glucose uptake and catalysis represent the primary sources of cellular energy production, while mitochondria are responsible for the majority of ATP generation through the tricarboxylic acid cycle (TCA) and the oxidative phosphorylation (OXPHOS) chain [4]. Reactive oxygen species (ROS) are primarily generated during glucose catabolism and are involved in various forms of regulated cell death, including pyroptosis, ferroptosis and necroptosis. Conversely, apoptosis is modulated by Cytochrome C and SMAC located in the mitochondrial intermembrane space (IMS) [5, 6]. In the realm of mitophagy and mitochondrial fusion and fission, mitochondrial dynamics dramatically regulate mitochondrial morphology, quantity and position within eukaryotic cells, which are essential for preserving the critical functions of mitochondria and cellular processes [7, 8]. Recent research has advanced our understanding of the relationship between mitochondria and tumor microenvironment (TME), revealing that mitochondria-related cellular metabolism, cell death and mitochondrial dynamics are closely linked to tumor immunosurveillance and antitumor immunity. These findings provide valuable insights into the potential application of immunotherapy in tumor treatment [9, 10].

Methionine metabolism is implicated in various cellular biological and molecular functions, including methylation reactions and redox maintenance, and plays a crucial role in tumor progression [11]. S-adenosylmethionine, a product of methionine metabolism, promotes RNA modifications and influences RNA splicing, nuclear export, stability and translation, thereby contributing to antitumor immunity [12, 13]. The types of RNA modifications, along with advanced, quantitative and sensitive transcriptome-wide sequencing techniques have been comprehensively detailed (Table 1 and 2). Recent discoveries have unveiled the intriguing regulatory mechanisms through which RNA modifications modulate glucose metabolism by controlling the translational efficacy of OXPHOS proteins, thereby facilitating tumor metastasis [14, 15]. Moreover, cuproptosis and antitumor immunity have been identified for the first time as being regulated by RNA modifications in malignancies, underscoring their clinical and research significance in tumor treatment [16, 17].

**Fig. 1:**
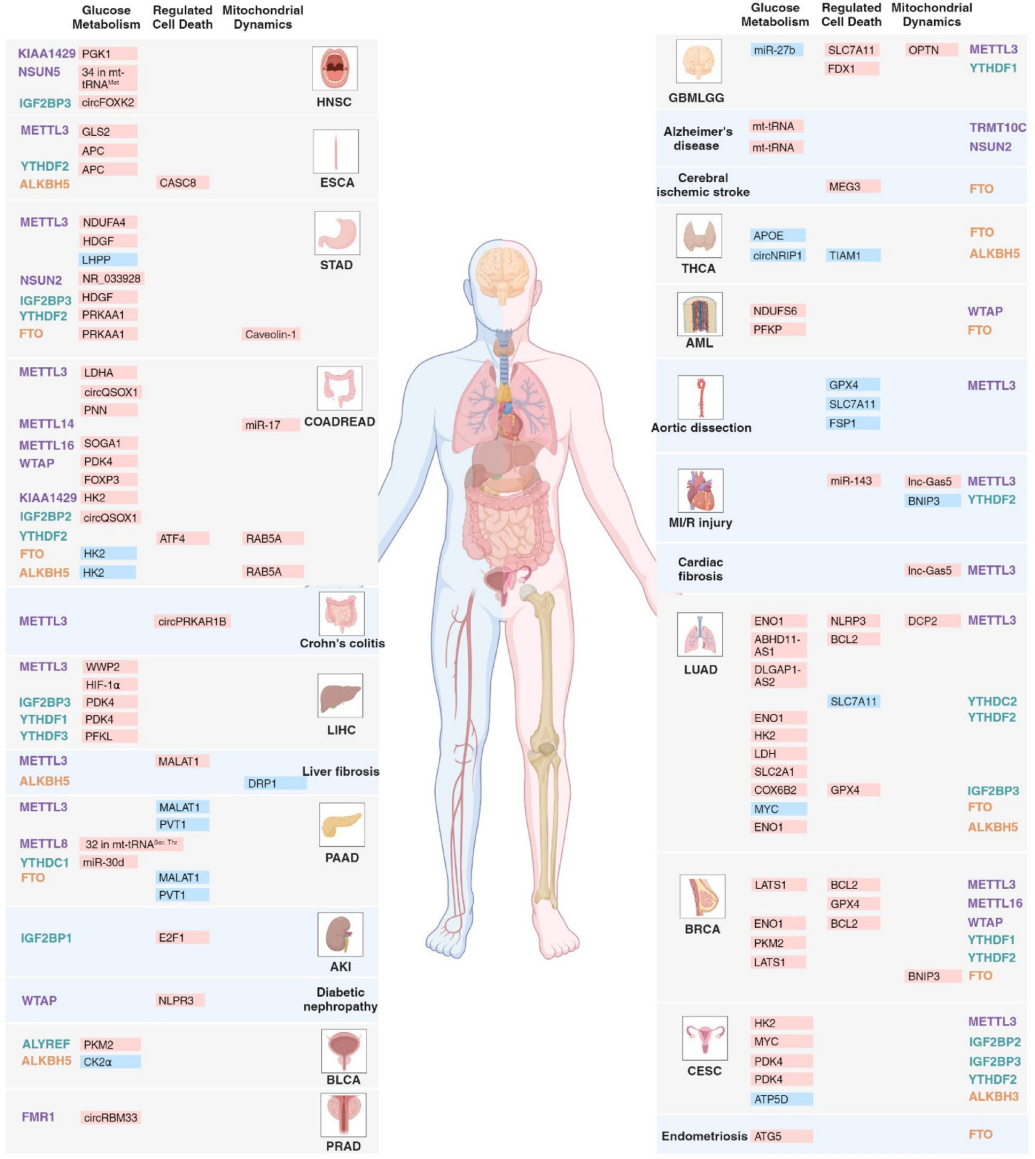
The role of mitochondria-related RNA modifications in cancer and non-cancer diseases. Disease-promoting (red) and disease-inhibiting (blue) roles of RNA modifications on downstream targets, including coding and non-coding RNAs, are listed for different cancer types (grey) and non-cancer diseases (blue). The associated genes are categorized on the basis of mitochondrial functions (glucose metabolism, regulated cell death and mitochondrial dynamics). RNA modifications modifiers are listed at the outer column, including writers (purple), readers (green) and erasers (yellow). GBMLGG: Low Grade Glioma and Glioblastoma, HNSC: Head and Neck cancer, THCA: Thyroid Cancer, LUAD: Lung Adenocarcinoma, LIHC: Liver Hepatocellular Carcinoma, AKI: Acute Kidney Injury, PAAD: Pancreatic Adenocarcinoma, PRAD: Prostate Adenocarcinoma, BLCA: Bladder Urothelial Carcinoma, ESCA: Esophageal Carcinoma, AML: Acute Myeloid Leukemia, BRCA: Breast Invasive Carcinoma, STAD: Stomach Adenocarcinoma, COADREAD: Colon Adenocarcinoma and Rectum Adenocarcinoma, CESC: Cervical Squamous Cell Carcinoma

**Fig. 2:**
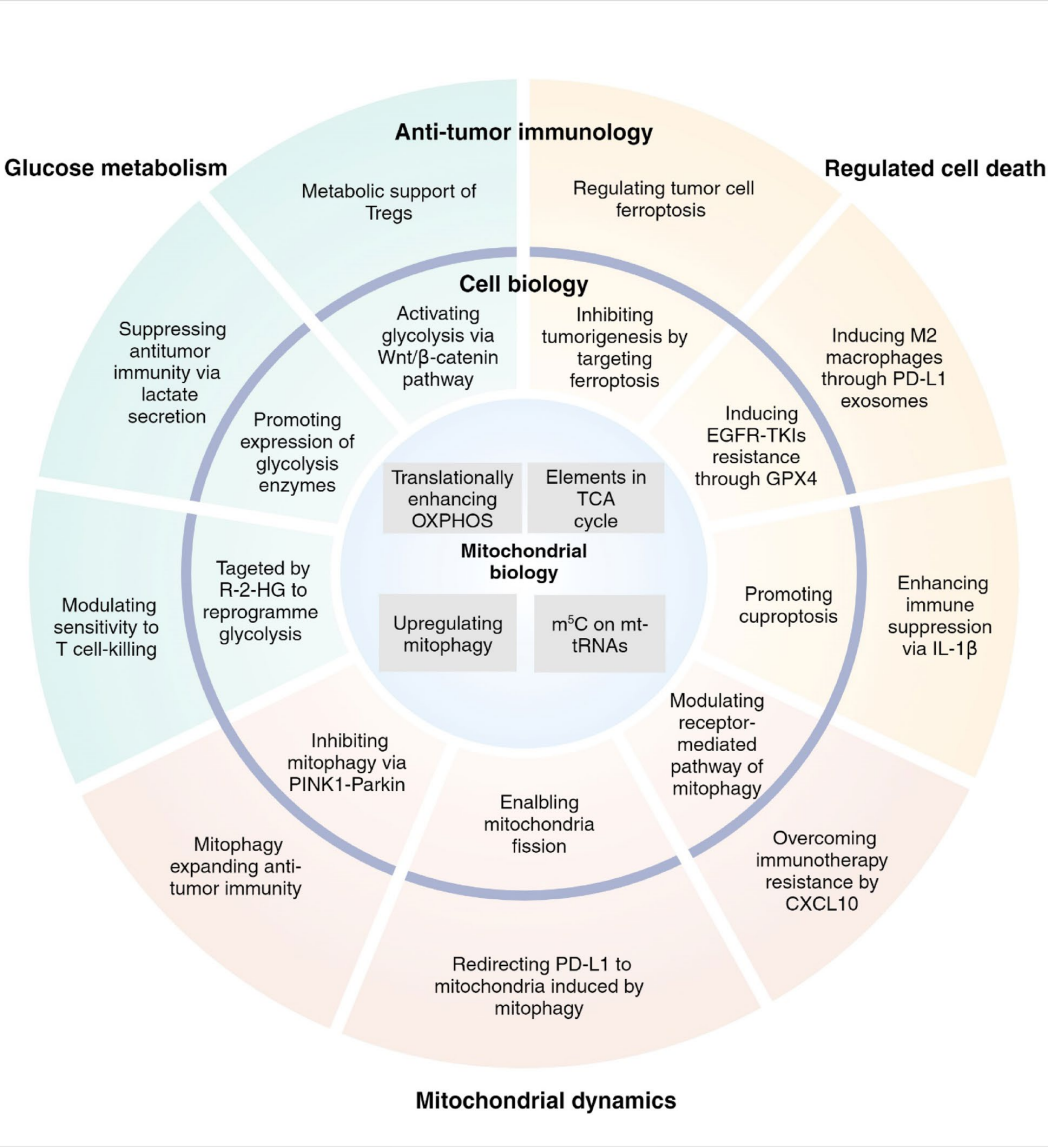
Hallmark findings of mitochondria-related RNA modifications. The hallmark findings are categorized on the basis of mitochondrial functions (glucose metabolism—green area, regulated cell death—yellow area and mitochondrial dynamics—red area) and into 3 levels (mitochondrial biology—inner circle, cell biology—median circle and antitumor immunology—outer circle)

A plethora of reviews have been published regarding the association between RNA modifications and diseases. However, only a limited number of these reviews focus on the subcellular level, particularly with mitochondria. RNA modifications exert both direct and indirect effects on the expression of genes involved in physiological and pathological processes associated with mitochondria, which holds significant value for elucidating specific mechanisms [1, 18]. In this review, we provide an overview of recent research concerning RNA modifications in mitochondria-related glucose metabolism, cell death, mitochondrial dynamics and antitumor immunity (Fig. 1), and briefly summarize the hallmark findings related to mitochondrial function and RNA modifications (Fig. 2). Additionally, we summarize small molecule inhibitors of RNA modification modifiers related to mitochondria, suggesting potential strategies for disease treatment.

## RNA modifications modifiers and their inhibitors

### RNA modifications modifiers

Depending on the specific cellular context of various diseases, alterations in RNA modifications and the dynamic changes in RNA modifiers----writers, readers and erasers----constitute a complex network of the cellular machinery that controls homeostasis and pathogenesis (Table 1) [19]. The deposition of m^6^A in mRNA is primarily facilitated by the m^6^A methyltransferase complex (MTC), which consists of three core components: methyltransferase-like 3 (METTL3), METTL14 and WTAP. In this complex, METTL3 serves as the principal catalytic subunit, METTL14 is responsible for substrate recognition, and WTAP aids in substrate recruitment [20–23]. Additionally, METTL16, key mediator of the N6-methyladenosine modification methyltransferase complex subunit (KIAA1429), along with METTL5 and ZCCHC4 have also been reported to possess m^6^A methyltransferase activity [24–27].

**Table 1:** RNA modifications modifiers related to mitochondrial biological process

Modification	Writer	Reported in mitochondrial biological process	Reader	Reported in mitochondrial biological process	Eraser	Reported in mitochondrial biological process
m^6^A	METTL3-METTL5-METTL14	METTL3-METTL5-METTL14 [28]	YTHDF1 to YTHDF3	YTHDF1 to YTHDF3 [19,29,30]	FTO	FTO [31]
	METTL16	METTL16 [32]	YTHDC1 and YTHDC2	YTHDC1 and YTHDC2 [33,34]	ALKBH5	ALKBH5 [15]
	KIAA1429	KIAA1429 [35]	IGF2BP1 to IGF2BP3	IGF2BP1 to IGF2BP3 [32,36,37]		
	WTAP	WTAP [38]	FMR1			
	ZCCHC4		HNRNPA2B1 and HNRNPC			
m^1^A	TRMT10C	TRMT10C [39]	YTHDF1		ALKBH3	ALKBH3 [40]
	TRM6-TRM61		YTHDF2		ALKBH1	
m^2^A	YFGB					
Am	FBL					
	CMTR1					
m^6^Am	PCIF1				FTO	
	METTL4					
m^6,2^A	DIM1					
m^5^C	NSUN1 to NSUN7	NSUN1 to NSUN7 [14,41,42]	ALYREF	ALYREF [43]	ALKBH5	
	DNMT2		YBX1		TET2	
m^3^C	METTL8	METTL8 [44,45]	SARS1		ALKBH3	
	METTL2		SARS2			
	METTL6		DALRD3			
m^7^G	METTL1					
	WDR4					
	WBSCR22					
	RNMT					
	RAM					
	TMRT112					

### Methodologies of RNA modifications profiling

Usage of transcriptome-wide profiling of RNA modifications by next-generation sequencing and high throughout techniques, such as MeRIP-seq, provides information on locus-specific changes with high resolution, which facilitates the exploration of the exact roles of RNA modifications of the target genes at an epitranscriptomic level (Table 2). GLORI and eTAM-seq, which were developed in the past 2 years, technically offer the opportunity for much higher resolution and specificity of quantitative transcriptome-wide profiling of RNA modifications at single-base level [46, 47]. However, more advanced methods are in need to provide more accurate, consistent and sensitive epitranscriptomic profiles of RNA methylation at both single-base and single-cell resolution.

**Table 2:** Detecting methodology of RNA modifications

Technique	Year	Description	Antibody based?	Resolution	Features	Limitations	Ref
m^6^A ELISA	1987	ELISA-based method to detect the amount of m^6^A	Yes	Not mentioned	Medium sensitivity	Lack of contaminations in normalization controls	[48]
Two-dimensional TLC	1995	Identifications of labeled nucleotide spots based on different chemical and physical properties followed by fluorescence quantification	No	Not mentioned	First known quantitative method	No qualitative information	[49–51]
meRIP-seq and meRIP-qPCR	2012	RNA immunoprecipitation depending on high specific antibodies followed by sequencing or qPCR	Yes	100-200nt	Can be used for any given modification	Cannot provide single-nucleotide resolution; Depends on immunoprecipitation efficiency	[52–54]
SCARLET	2013	RNase H site-specific cleavage, splinted ligation, ribonuclease digestion, TLC, P32 labeling to quality methylations	No	Single nucleotide	Quantifying modifications in individual mRNA/lncRNA at single-nucleotide resolution	Only assesses one specific site per assay	[55]
PA-m^6^A-seq	2015	Photo-crosslinking-assisted strategy applied to high-resolution mapping of m^6^A	Yes	~ 23 nt	Improved resolution compared with m^6^A-seq/MeRIP-seq	Cannot provide single-nucleotide resolution; antibody-related limitations	[56]
miCLIP	2015	m^6^A individual-nucleotide-resolution cross-linking and immunoprecipitation	Yes	Single nucleotide	Identifying precise m^6^A positions on a transcriptome-wide level at single-nucleotide resolution	Large amount of starting material required	[57]
m^6^A-LAIC-seq	2016	Quantitatively deconvolutes methylated versus nonmethylated transcripts relying on sequencing intact full-length transcripts in both m^6^A-positive and m^6^A-negative fractions post-RIP	Yes	Full-length transcript	Quantifying the ratio of methylated to nonmethylated transcripts on a transcriptome-wide scale	Dependent on antibody specificity	[58]
HPLC	2016	High-performance liquid chromatography coupled with mass spectrometry	No	Single nucleotide	Measurement of a large number of modifications at the same time	Unable to detect modifications on long RNAs	[59]
LC-MS/MS	2017	Digestion to nucleoside level followed by liquid chromatography and mass spectrometer measurement	No	Not mentioned	Highly quantitative	Contaminating mycoplasma or digestion enzymes may affect methylated bases level	[60]
Nm-seq	2017	Leveraging oxidative cleavage of ribose 2′,3′-vicinal diols by periodate to expose, enriching and mapping Nm sites in the transcriptome	No	Single nucleotide	Nucleoside resolution, more sensitive than RiboMeth-seq	Depending on differential reactivity of 2′-OMe versus 2′-OH nucleotides toward periodate oxidation	[61]
SELECT	2018	Hindering elongation activity of DNA polymerases and the nick ligation efficiency followed by qPCR quantification	Yes	Single nucleotide	Highly sensitive at specific sites	Dependent on specific antibodies	[62]
SMRTseq, nanopores and xPore	2018	Identifying positions of m^6^A sites at single-base resolution and quantifying the differential modification rate across conditions	No	Single nucleotide	Enabling simultaneous profiling of differential transcript expression and modification	High error rates if not optimized for appropriate base detection	[63, 64]
m^6^A RT-QPCR	2019	Taking advantage of the diminished capacity of BstI enzyme to retrotranscribe m^6^A residues for the relative quantification of candidate m^6^A regions	Yes	Not mentioned	Relatively short and independent of NGS	Dependent on specific antibodies	[65]
m^6^ACE-seq	2019	M^6^A crosslinking exonuclease sequencing to map transcriptome-wide m^6^A	Yes	Single nucleotide	High resolution with a comprehensive atlas of distinct methylomes uniquely mediated by every individual known methyltransferase or demethylase	Relying on exonuclease specificity and efficiency; antibody-related limitations	[66]
DART-seq and scDART-seq	2019	Cytidine deaminase APOBEC1-YTH expression in cells induces C-to-U deamination adjacent to m6A residues followed by scRNA-seq	No	Single nucleotide	Highly sensitive for single-cell analyses; Ultra-low RNA input	Lacks quantitative information; detects only YTH-binding m6A sites	[67, 68]
MAZTER-seq and m^6^A-REF-seq	2019	A bacterial single-stranded RNase MazF to digest cellular RNAs at unmethylated ACA sites followed by MAZTER-seq to quantify m^6^A methylations	No	Single nucleotide	Antibody-independent method at single-nucleotide resolution; high specificity	Limited on detecting methylation at the specific sites MazF captured; detects only m6ACA motif due to enzyme specificity; detect only the 16–25% of total m^6^A sites	[69, 70]
m^6^A-SEAL	2020	Depending on the ability of FTO RNA demethylase to convert m^6^A into a reactive intermediate followed by tagging and sequencing	No	~ 200nt	Highly sensitive, low false-positive rates and flexible for detection of other RNA modification	Low resolution; depending on FTO oxidation efficiency of m^6^A;	[71, 72]
m^6^A-label-seq	2020	Feeding cells an analog of methionine leading to replacement of mRNA m^6^A forming sites to the N6-allyladenosine (a6A), followed by mis-incorporations during RT and sequencing	Anti-a 6 A antibody-dependent	Single nucleotide	High resolution; better at detecting clustered m6A sites	Low a6A labelling efficiency; suitable for only in vitro studies	[73, 74]
meCLICK-seq	2020	Click-degraders that attacheto RNA species through click-chemistry and can degrade them, hijack RNA methyltransferase activity to introduce an alkyne, instead of a methyl	No	Single nucleotide	High resolution at intronic and intergenic regions	Detection is depletion, not enrichment, of modified RNA species	[75]
SLIM-seq	2022	Decoding m^6^A modification on full-length transcript at the expense of the regional information of m^6^A peaks	Yes	Full-length transcript	Highly sensitive and efficient for rare cells	Low resolution without absolute stoichiometric information at single nucleotide level	[76]
m^6^A-SAC-seq	2022	m^6^A-selective allyl chemical labeling and sequencing from cell line	No	Single nucleotide	Requires only ~ 30 ng of poly(A) or rRNA-depleted RNA; First method offering single-base m^6^A stoichiometries	Significant preference of GAC over AAC motif	[77]
GLORI	2023	Glyoxal and nitrite-mediated deamination of unmethylated adenosines followed by RNA sequencing	No	Single nucleotide	High-throughput, single-base-resolution method	Unable to differentiate m^6^A from m^6^Am/m^1^A	[46]
eTAM-seq	2023	Enzyme-assisted sequencing technology that detects and quantifies m^6^A by global adenosine deamination	No	Single nucleotide	Requires low RNA input with as few as ten cells for m^6^A quantification	Less sensitive to lowly methylated sites	[47]

### Inhibitors of RNA modification modifiers

Inhibitors of m^6^A writers are emerging (Table 3). In 2021, Yankova E et al. reported a highly potent and selective first-in-class catalytic inhibitor of METTL3, STM2457, which inhibited AML development by selectively reducing m^6^A levels and the expression of bromodomain protein 4 (BRD4) mRNA [78]. Substantial research has confirmed the antitumor role of STM2457 across multiple tumors, including AML, SCLC, NSCLC, hepatocarcinoma (HCC), pancreatic cancer, medulloblastoma, osteosarcoma and intrahepatic cholangiocarcinoma [79–86]. STM2457, which suppresses BRD4 RNA modifications, significantly impacts mitochondrial glucose metabolism, ferroptosis and mitochondrial dynamics. Another gene targeted by STM2457, DCP2, is strongly associated with mitophagy [79]. Other METTL3 inhibitors, including Cpd-564, UZH1a and UZH2, have been developed in recent years and have demonstrated promising preclinical efficacy in the treatment of AKI, AML and prostate cancer [87–89]. Furthermore, a phase 1 study (NCT05584111) of STC-15, another METTL3 inhibitor, in advanced malignancies is currently underway.

Readers of m^6^A can be categorized into three primary classes: YT521-B homology (YTH) domain family proteins, which include YTHDF1–3 and YTHDC1–2, insulin-like growth factor 2 mRNA-binding proteins (IGF2BPs), comprising IGF2BP1–3, and heterogeneous nuclear ribonucleoproteins (hnRNPs), including hnRNPA2B1, hnRNPC and hnRNPG. These proteins recognize m^6^A modifications on target RNAs and mediate various biological processes, including RNA stability, translational efficiency, cellular localization and RNA splicing [12].

Inhibitors of m^6^A readers have been meticulously developed recently (Table 3). Notably, BTYNB, an inhibitor of IGF2BP1, has been shown to exert antiproliferative effects in IGF2BP1-expressing ovarian cancer and melanoma cells, while exhibiting no effects in IGF2BP1-negative cells [90]. Feng P et al. demonstrated that JX5 reduced the m^6^A modification level of NOTCH1 mRNA through the inhibition of IGF2BP2, subsequently decreasing NOTCH1 expression and indicating a significant therapeutic effect on T-ALL [36]. Another IGF2BP2 inhibitor, CWI1-2, effectively inhibits leukemia in vivo by targeting MYC, glutamate pyruvate transaminase 2 (GPT2) and SLC1A5 mRNAs, leading to a drastic downregulation of mitochondria-related glutamine metabolic pathways and energy production [91].

Two erasers, the α-ketoglutarate (αKG)-dependent dioxygenases FTO and alkB homolog 5 (ALKBH5) have been identified as responsible for the demethylation of m^6^A-modified RNAs, which regulate targeted gene expression [92, 93]. FTO exhibits a broader substrate range, acting on multiple methylation sites, but demonstrates a significantly higher catalytic efficiency for m^6^Am, particularly those located near the 5′ cap of mRNA [94]. In contrast, ALKBH5 shows a more refined sequence preference, primarily recognizing substrate motif Pu[G >A]m^6^ACH, with a strong bias for the ACm^6^ACH and GCm^6^ACH sequences [93]. This specificity suggests that ALKBH5 may target distinct subsets of transcripts, influencing their half-life and splicing.

A series of small-molecule inhibitors targeting FTO have been developed demonstrating promising therapeutic activity across several diseases, including AML, clear cell renal cell carcinoma, nasopharyngeal carcinoma, hepatocarcinoma, breast cancer, glioblastoma, glioma, esophageal cancer, melanoma, gastric cancer, uterine leiomyosarcoma, obesity, epilepsy, Alzheimer’s disease, pancreatitis and diabetes (Table 3) [31, 95–122]. Notably, R-2-hydroxyglutarate (R-2HG) has been shown to inhibit FTO activity, which enhances m^6^A modification and decreases the stability of MYC/CEBPA mRNAs, leading to the downregulation of critical pathways, including glucose metabolism and mitochondria-related cell death [104]. Additionally, one study revealed that R-2HG attenuated aerobic glycolysis in leukemia by targeting the FTO/m^6^A/PFKP/LDHB axis [31]. Importantly, FB23-2, a small-molecule inhibitor of FTO, selectively inhibits the m^6^A modification of ankyrin repeat and SOCS box containing 2 (ASB2) mRNA, resulting in the upregulation of ASB2, which decreases mitochondrial membrane potential, thereby triggering intracellular signaling pathways that culminate in cytoplasmic vacuolization and paraptotic cell death [95, 123]. Another small-molecule inhibitor of FTO, CS1/CS2, suppresses cancer stem cell maintenance and immune evasion by targeting FTO activity on MYC and CEBPA mRNAs [100]. Furthermore, 18097, another FTO inhibitor, enhances the mRNA stability of SOCS1, which facilitates BAX recruitment to mitochondria and induces apoptosis [101, 124]. Saikosaponin D, GNPIPP12MA and ZLD115a, three novel FTO inhibitors, exhibit exceptional efficacy in inhibiting FTO’s activity on MYC mRNA, downregulating aerobic glycolysis and suppressing tumor progression [105, 106, 115].

Small-molecule inhibitors of ALKBH5 have been developed in recent years and have been shown to be effective in the treatment of various diseases, including AMI, AKI, AML, melanoma and glioblastoma (Table 3) [125–132]. In particular, ALK-04 inhibits the ALKBH5-mediated upregulation of MCT4, leading to reduced lactate export to the TME and increased efficacy of cancer immunotherapy [127]. Another ALKBH5 inhibitor, DDO-2728, has been demonstrated to increase the abundance of m^6^A modifications in AML cells and inhibit cell cycle progression via a reduction in the mRNA stability of *TATC3* [128]. The gene fusion of fibroblast growth factor receptor 3 (FGFR3)-TATC3 was shown to activate OXPHOS and mitochondrial biogenesis [133]. Furthermore, Ena15/Ena21 inhibits the cell proliferation of glioblastoma multiforme-derived cell lines, increases m^6^A levels and stabilizes forkhead box M1 (FOXM1) mRNA [130]. FOXM1 can inhibit OXPHOS by translocating to mitochondria and upregulating the pentatricopeptide repeat domain 1 (PTCD1) protein, a mitochondrial leucine-specific tRNA binding protein that inhibits leucine-rich ETC complexes, or by binding to mtDNA and suppressing mitochondrial activity [134, 135].

Inhibitors of m^5^C modifiers are emerging. Nsun2-i4, a small-molecule inhibitor of NSUN2, downregulates the m^5^C modification on ENO1 mRNA, leading to reduced glycolysis and enhanced efficacy of cancer immunotherapy [136]. Methodology of cysteine-directed activity-based protein profiling (ABPP) discovers azetidine acrylamides as covalent inhibitors of NSUN2 that stereoselectively react with the catalytic C271, while showing negligible cross-reactivity with other NSUNs in human cells [137]. Azetidine acrylamides disrupt NSUN2-tRNA interactions in cancer cells, leading to a global reduction in tRNA m^5^C content, thus mediating mitochondrial functions. Pharmacological inhibition of NSUN2 with MY-1B exhibits potent anti-leukemic effects, synergizing robustly with ferroptosis inducers, standard chemotherapy, and the BCL-2 inhibitor venetoclax [138].

Small-molecule inhibitors of m^1^A modifiers have been developed in recent years. The ALKBH3 inhibitor HUHUS015 disrupts the demethylation of HK2 via m^1^A, resulting in inactivated glycolysis and reduced lactate production [40]. Moreover, HUHUS015 inhibits choroidal neovascularization (CNV) synergistically with the anti-VEGF drug Aflibercept [40]. Another ALKBH3 inhibitor, compound 7I exhibits more potent inhibitory activities than that of HUHS015 in vivo without negative side-effects [139]. The first potent and isoform selective inhibitor of ALKBH1 has been discovered as 13 h, which is able to engage ALKBH1 and modulate the m^1^A levels [140]. Another highly potent ALKBH1 inhibitor, 1*H*-pyrazole-4-carboxylic acid derivative 29E, inhibits cell viability, and upregulated the AMPK signaling pathway [141].

Furthermore, modifiers of other forms of RNA modifications, including m^1^A, m^2^A, Am, m^6^Am, m^6,2^A, m^5^C, m^3^C and m^7^G, have been comprehensively reviewed (Table 1).

**Table 3:** Small-molecule inhibitors targeting RNA methylation modifiers

Target	Inhibitor	Disease	Modified gene related to mitochondria	Role	Ref
FTO	FB23-2	AML	ASB2	Increased mitochondrial membrane potential	[95, 123]
		Clear cell renal cell carcinoma	SIK2	Decreased ROS production; Downregulation of PINK1-Parkin-mediated mitophagy	[96, 142, 143]
		Nasopharyngeal carcinoma	OTUB1	Upregulation of ferroptosis	[97]
		AML	-	-	[98]
		Hepatocellular carcinoma	-	-	[99]
	CS1/CS2	AML	MYC	Downregulation of glucose metabolism	[100]
			CEBPA	Downregulation of OXPHOS	[144]
	18,097	Breast cancer	SOCS1	Upregulation of apoptosis	[101, 124]
	MA	Glioblastoma	-	-	[102, 103]
	R-2HG	AML; Glioma	MYC/CEBPA	Downregulation of glucose metabolism	[104]
		AML	PFKP	Downregulation of glycolysis	[31]
	Saikosaponin D	AML	MYC	Downregulation of glucose metabolism	[105]
	Radicicol	AML	-	-	[107]
	N-CDPCB	Obesity	-	-	[108]
	MO-I-500	Epilepsy	-	-	[109]
		Alzheimer’s disease	-	-	[110]
		Breast cancer	-	-	[111]
	Nafamostat mesylate	Pancreatitis	-	-	[113]
	CHTB	Obesity	-	-	[112]
	C6	Esophageal cancer	-	-	[114]
	ZLD115	AML	MYC	Downregulation of glucose metabolism	[115]
	Dac51	Melanoma	-	-	[116]
		Diabetes	-	-	[117]
		Uterine leiomyosarcoma	-	-	[118]
	Rhein	Breast cancer	-	-	[119]
	FTO-04	Glioblastoma	-	-	[120]
	FTO-43	AML; Glioblastoma; Gastric cancer	-	-	[121]
	MU06	Obesity	-	-	[122]
METTL3	STM2457	AML	BRD4	Downregulation of ferroptosis, fatty acid and glucose metabolism, mitochondrial fission and fusion; Upregulation of apoptosis	[78, 145–153]
		SCLC	DCP2	Upregulation of PINK1-Parkin-mediated mitophagy	[79]
		HCC	-	-	[81]
		Pancreatic cancer	-	-	[82]
		NSCLC	-	-	[83]
		Osteosarcoma	-	-	[85]
		Intrahepatic cholangiocarcinoma	NFAT5	Downregulation of glycolysis	[86]
	Cpd-564	AKI	-	-	[87]
	UZH1a	AML	-	-	[88]
	UZH2	AML; Prostate cancer	-	-	[89]
IGF2BP2	JX5	T-ALL	NOTCH	-	[36]
	CWI1-2	AML	MYC	Downregulation of glucose metabolism	[91]
			GPT2	Downregulation of TCA cycle	[154, 155]
YTHDF1-2	Ebselen	Prostate cancer	-	-	[156]
ALKBH5	IOX1	AMI	-	-	[125]
		AKI	-	-	[126]
	ALK-04	Melanoma	MCT4	Downregulation of lactate secretion	[127]
	DDO-2728	AML	TACC3	Downregulation of mitochondrial biogenesis	[128, 133]
	Cpd-20m	-	-	-	[129]
	Ena15/Ena21	Glioblastoma	FOXM1	Upregulation of ETC, mitochondrial activity; Downregulation of glycolysis	[130, 134, 135, 157]
	RD3/RD6	AML	-	-	[131]
	MV 1035	Glioblastoma	-	-	[132]

## RNA modifications in mitochondria-related glucose metabolism

Through a series of biochemical reactions catalyzed by three key enzymes, hexokinase 2 (HK2), phosphofructokinase (PFK) and pyruvate kinase (PK), glucose is transported from the extracellular fluid via the glucose transporter (GLUT) and is converted into pyruvate through glycolysis in the cytosol [3]. This process is followed by the TCA cycle in the mitochondria matrix under normoxia, with the electrons generated above subsequently flowing through the ETC and drive OXPHOS. Conversely, during hypoxia, mitochondrial OXPHOS is suppressed, leading to lactate generation catalyzed by lactate dehydrogenase(LDH) [158, 159]. In tumor cells, glycolysis, rather than mitochondrial respiration, predominates for energy production, resulting in excessive lactate production and enhanced tumor progression [160].

Elucidating the precise function of RNA modifications in glycolysis is essential. This is of paramount importance because the glycolytic pathway directly provides the metabolic intermediates, such as pyruvate, that fuel subsequent mitochondrial metabolism, including the TCA cycle and OXPHOS. Many diseases, including cancer and metabolic disorders, are characterized by a loss of metabolic flexibility—the cell’s ability to switch between using glycolysis and OXPHOS. Consequently, a comprehensive understanding of RNA modification’s influence on glycolysis is a prerequisite for fully grasping its regulatory impact on mitochondrial-related glucose metabolism.

**Fig. 3:**
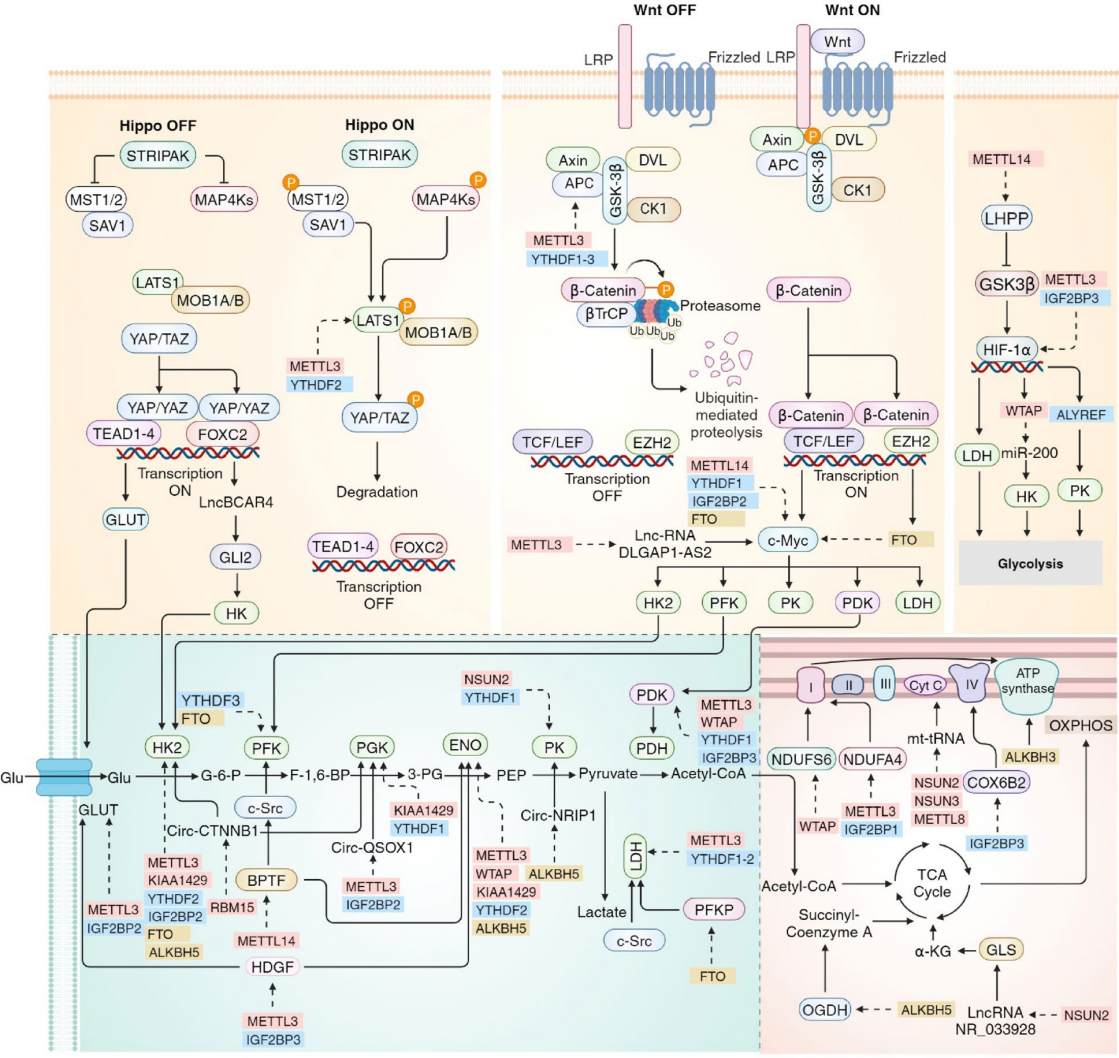
RNA modifications in mitochondria-related glucose metabolism. Glucose is metabolized via glycolysis in the cytosol (green area), TCA cycle and OXPHOS in mitochondria (red area). The Hippo, Wnt and HIF-1α pathways regulate the expression levels of enzymes that participate in glycolysis. RNA modifications in mitochondria-related glucose metabolism is implemented by RNA modifications modifiers, including writers in red, readers in blue and erasers in yellow

### RNA modifications in signaling pathways that regulate mitochondria-related glycolysis

The RNA modifications of target transcripts play a crucial role in modulating the expression of key molecules involved in various glucose metabolism-related signaling pathways, including the Hippo, Wnt and HIF-1α pathways (Fig. 3). Recently, Xu Y et al. reported that METTL3 cooperates with YTHDF2 to promote the modification of large tumor suppressor kinase 1 (LATS1) mRNA, thereby reducing its stability. This reduction leads to the upregulation of Yes-associated protein 1/WW-domain-containing transcription regulator 1 (YAP/TAZ) and subsequently enhances the transcriptional activity of associated domain (TEAD1-4) and the forkhead box C2 (FOXC2), which regulate the expression of GLUT3 and lncRNA breast cancer anti-estrogen resistance 4 (BCAR4) [162, 163]. This cascade of events ultimately promotes glycolysis and facilitates the aerobic respiration process in mitochondria, contributing to breast cancer tumorigenesis [19].

As one of the target genes regulated by T cell factor/lymphoid enhancing factor (TCF/LEF) in the WNT signaling pathway, *MYC* plays an essential role in the regulation of glucose metabolism, transcriptionally mediating the expression of target genes including GLUT1, HK2, PFK, PKM2, PDK and LDHA, which has been demonstrated to be modulated by RNA modifications [164] (Fig. 3). Aerobic glycolysis in cervical cancer can be regulated by m^6^A-MYC expression through the METTL14-MYC-IGF2BP2-FTO axis [165]. In non-small cell lung cancer (NSCLC), METTL3 enhances the stability of the lncRNA DLGAP1 antisense RNA 2 (DLGAP1-AS2), which interacts with YTHDF1 and promotes c-MYC mRNA stability via m^6^A modification [29]. Moreover, TRMT61A-mediated tRNA-m^1^A modification promotes MYC protein synthesis, upregulating PD-L1 expression and suppressing antitumor immunity [166]. The upregulation of glycolysis through c-Myc further facilitates the TCA cycle and OXPHOS in mitochondria.

Regarding the HIF-1α pathway, METTL3 can enhance the transcription of LDHA indirectly through m^6^A modification of HIF-1α mRNA in conjunction with IGF2BP3 recruitment [167]. Furthermore, under hypoxia, HIF-1α positively regulates WTAP, which regulates the expression of microRNA-200 (miR-200) and HK2, significantly accelerating the intracellular Warburg effect [38]. Wang JZ et al. identified an m^5^C-dependent modification of PKM2 mRNA following the HIF-1α/Aly/REF export factor (ALYREF)/PKM2 axis, promoting glucose metabolism in bladder cancer [43]. Importantly, HIF-1α can interact with the Wnt/β-catenin signaling pathway through the transcriptional upregulation of calreticulin (CALR), thereby enhancing tumor development [168]. Moreover, FTO reduces the m^6^A modification level of NPAS2 in macrophages and mediates inflammation and glycolysis in M1 macrophages by regulating the HIF-1α signaling pathway, leading to suppressed macrophages inflammation and glycolysis in diabetic nephropathy [169].

### RNA modifications in the regulation of glycolysis components and enzymes

METTL3 directly interacts with the 3’UTR of GLUT1 via IGF2BP2/3 and increases the stability of GLUT1 mRNA in cooperation with circFOXK2, upregulating cell glycolysis and the subsequent TCA cycle in mitochondria [28, 170]. The lncRNA LINC00958 is m^6^A modified by KIAA1429 and upregulated, followed by interaction with GLUT1 mRNA in a m^6^A-dependent manner [35]. In pancreatic ductal adenocarcinoma, YTHDC1 mediates miR-30d mRNA stability in a m^6^A-dependent manner and facilitates its maturation [171]. Moreover, miR-30d inhibits glycolysis and tumor progression through downregulating GLUT1 and HK1 expression by directly targeting the transcription factor RUNX1. Suppression of FTO significantly stabilizes apolipoprotein E (APOE) mRNA and coordinates with IGF2BP2, leading to the promotion of GLUT1 expression and tumor glycolysis by modulating the IL-6/JAK2/STAT3 signaling pathway [172]. The m^6^A modification of WW domain-containing protein 2 (WWP2) mRNA by METTL3 and IGF2BP2 increases WWP2 level, which stimulates the AKT signaling pathway and hence enhances glycolysis and OXPHOS in mitochondria via the upregulation of GLUT1 [173–175]. Furthermore, the NSUN2-mediated GLUT1 stabilization via m^5^C modification enhances the competitive advantage of tumor cells in glucose acquisition, accelerating malignancy in HCC [41].

HK2 can be directly mediated by the m^6^A modifiers METTL3, YTHDF1, IGF2BP2, FTO and ALKBH5 in colorectal cancer and cervical cancer, leading to increased glycolysis, upregulated aerobic respiration in mitochondria and tumor progression [170, 176–179]. The m^6^A modification of WWP2 can also enhance glycolysis through the activation of the AKT signaling pathway, thereby upregulating HK2 [174, 175]. The m^1^A eraser ALKBH3 demethylates HK2 to activate glycolysis, resulting in excess lactate production. This lactate promotes histone lactylation at H3K18, which in turn bound to ALKBH3 to amplify its transcription, establishing a positive feedback loop [40]. Another positive functional loop exists between YTHDF3 and phosphofructokinase liver (PFKL), as YTHDF3 suppresses PFKL mRNA degradation via m^6^A modification, whereas PFKL positively regulates YTHDF3 expression by inhibiting the ubiquitination of the YTHDF3 protein through interaction with elongation factor Tu GTP binding domain containing 2 (EFTUD2), a core subunit of the spliceosome involved in the pre-mRNA splicing process [30, 180, 181]. Furthermore, YBX1 ensures the stability of PFKFB4 mRNA by recognizing m^5^C sites in its 3’UTR, resulting in upregulated glycolysis and enhanced LUSC development [182]. ALKBH3 positively regulates aldolase A (ALDOA) mRNA stability through m^1^A demethylation at the 3’UTR, leading to upregulated glycolysis and reduced overall survival in triple-negative breast cancer patients [183]. In oral squamous cell carcinoma (OSCC), m^6^A modification of PGK1 mRNA can be facilitated by KIAA1429 and recognized by YTHDF1, resulting in increased stability of PGK1 mRNA [184]. Bioinformatic analyses revealed that through METTL3 and IGF2BP2, circQSOX1 was upregulated and sponged with miR-326 and miR-330-5p to promote PGAM1 expression, which further activated glycolysis and cytotoxic T-lymphocyte-associated antigen-4 (CTLA-4) expression, contributing to tumor immune escape [185]. ENO can be modulated by m^6^A modifiers, including METTL3, KIAA1429, WTAP, YTHDF2 and ALKBH5, mediating the cell glycolysis process in lung adenocarcinoma, breast cancer and ovarian cancer [186–189]. Another study indicated that overexpression of FTO increased autophagy related 5 homolog (ATG5) protein expression at low m^6^A levels, reduced PKM2 expression levels, and decreased mitochondrial ATP production in ectopic endometriotic stromal cells [190]. Qi et al. reported that NSUN2 could stabilize PKM2 mRNA by increasing the m^5^C level in the 3’ UTR of PKM2 mRNA, promoting glycolysis and the progression of HCC [191]. In colorectal cells, LDHA mRNA can be directly modified by METTL3, YTHDF1 and YTHDF2, resulting in upregulated LDHA and lactate levels, as well as increased chemotherapy resistance [167]. The FTO/PFKP/LDHB axis has been shown to be involved in glycolysis in leukemia via m^6^A modification [31]. Furthermore, NSUN2 mediates m^5^C modification of tRNA^Val−CAC^, enhancing the codon-frequency-dependent translation of key glycolysis-related genes, including ALDH3A2, ALDH7A1, HK1, and PFKM, ultimately advancing glycolysis and TNBC cell proliferation, migration, and invasion [192].

### RNA modifications in the regulation of the TCA cycle and OXPHOS

Pyruvate is converted into acetyl-CoA and enters into the TCA cycle via pyruvate dehydrogenase (PDH), which is phosphorylated by pyruvate dehydrogenase kinase (PDK). However, the inconsistent roles of RNA modifications on PDK mRNA have been reported, necessitating further investigation. METTL3-, YTHDF1- and IGF2BP3-dependent m^6^A modification on the 5’UTR of PDK4 mRNA positively regulates its translation elongation and mRNA stability, whereas WTAP-mediated m^6^A modification on PDK4 mRNA downregulates PDK4 expression levels and facilitates tumor cell progression, indicating opposing effects of m^6^A modifications on PDK mRNA [27, 193]. Additionally, METTL16 collaborates with IGF2BP1 to enhance the expression of the suppressor of glucose by autophagy (SOGA1), which subsequently promotes the ubiquitination of AMP-activated protein kinase (AMPK) complex, ultimately resulting in the upregulation of PDK4 [32]. PDH can also be modulated in a m^6^A-dependent manner that is independent of PDK. METTL3-methylated circRBM33 interacts with fragile X messenger ribonucleoprotein 1 (FMR1), stabilizing PDHA1 mRNA and activating mitochondrial metabolism [194]. In colon adenocarcinoma, WTAP and YTHDF1 stabilize forkhead box P3 (FOXP3) mRNA, which further binds to the SMARCE1 promoter for transcriptional activation. This activation stimulates the MAPK signaling pathway, inducing the phosphorylation of cytosolic PDHE1α at S327 by ERK2 and its translocation to mitochondria, thereby advancing the TCA cycle in mitochondria and increasing ROS production, resulting in enhanced resistance to cytotoxic immune cells [195, 196]. Furthermore, NSUN2-mediated m^5^C modifications of the lncRNA NR-033928 interact with IGF2BP3 to promote glutaminase (GLS) mRNA stability, leading to increased production of glutamate and α-KG, along with succinyl-coenzyme A, which is catalyzed by α-ketoglutarate dehydrogenase (OGDH) demethylated by ALKBH5, collectively accelerating the TCA cycle rate and tumor development [15, 197].

RNA modifications are also involved in the regulation of the OXPHOS. NADH: ubiquinone oxidoreductase subunit S6 (NDUFS6), a subunit of the first complex in the ETC, is modulated by WTAP. Another subunit of complex I of the ETC, NDUFA4, can be regulated by METTL3 and IGF2BP1, resulting in increased glycolysis, oxidative metabolism and gastric cancer cell progression [198, 199]. Lin Z et al. revealed that metabolic reprogramming has been observed in NSCLC through RNA modifications of cytochrome c oxidase subunit 6B2 (COX6B2) via the IGF2BP3-COX6B2 axis, which could alter OXPHOS [37]. ATP synthase is regulated by the m^1^A demethylase ALKBH3, which negatively regulates ATP synthase translation elongation by increasing its binding with the YTHDF1/eRF1 complex, thereby facilitating the release of mRNA from the ribosome complex [200]. Posttranscriptional modification of mitochondrial tRNA is critical for the translational regulation of mitochondrial respiratory chain proteins. m^5^C at position 34 in mt-tRNA^Met^ modified by NSUN3, m^5^C at positions 48–50 in mt-tRNA^Tyr^, mt-tRNA^His^, mt-tRNA^Leu (UUR)^, mt-tRNA^Phe^ and mt-tRNA^Glu^ modified by NSUN2, and m^3^C at position 32 of the mt-tRNA^Ser (UCN)^ and mt-tRNA^Thr^ modified by METTL8 have been discovered to be closely associated with the translational efficacy of ETC proteins and mitochondrial respiratory function [14, 44, 45, 201]. The assembly and maturation of mitochondrial complexes are jointly orchestrated by tRNA-guided translation and complex assembly factors, such as cytochrome c oxidase assembly protein 18 (COX18) and cytochrome c oxidase assembly factor 1 (COA1), which collectively regulate the quality control of both mitochondrially-encoded and nuclear-encoded components [202, 203]. Moreover, RNA modifications of mitochondrial rRNA, including m^6,2^A modifications in helix 45 (h45) of 12 S mt-rRNA modified by mitochondrial transcription factor B1 (TFB1M), m^4^C modification at position C840 and m^5^C modification at position C842 of 12S mt-rRNA by METTL17 and m^4^C at position C839 of 12S mt-rRNA by METTL15, dominate the translational function of mitochondria-coding genes that influence mitochondrial respiration and other biological processes [204–206].

RNA modifications play an important role in the dynamic interaction among glycolysis, the TCA cycle, and OXPHOS. Overexpressed hnRNPCL2 recruits to Drosha/DiGeorge syndrome critical region 8 (DRG8) complexes and promotes a subset of miRNAs processing, including miR-483, miR-877, and miR-676, in a m^6^A dependent manner. The upregulated miRNAs inhibit OXPHOS and promotes glycolysis by targeting key players in the mitochondrial respiration, leading to a mitochondrial metabolic switch and cell proliferation in CRC [207]. NSUN1-mediated m^5^C modification inhibits c-Myc mRNA degradation and increases the expression of the glycolytic genes LDHA, PKM2, and ENO1, which leads to enhanced glycolysis and reduced OXPHOS on HCC cells [208]. Moreover, NSUN2 mediates m^5^C modification of tRNA^Val−CAC^, enhancing the codon-frequency-dependent translation of key glycolysis-related genes, including HK1 and PFKM, orchestrating the metabolic reprogramming between glycolysis and OXPHOS, leading to advanced chemoresistance in TNBC [192]. Furthermore, METTL1 drives a metabolic shift from glycolysis to OXPHOS through m^7^G tRNA modification in anlotinib-resistant OSCC [209].

Overall, further investigations into the role of RNA modifications in glucose metabolism, utilizing various RNA modification profiling methods are essential for providing a more comprehensive understanding and identifying potentially valuable targets for disease treatment.

## RNA modifications in mitochondria-related cell death

**Fig. 4:**
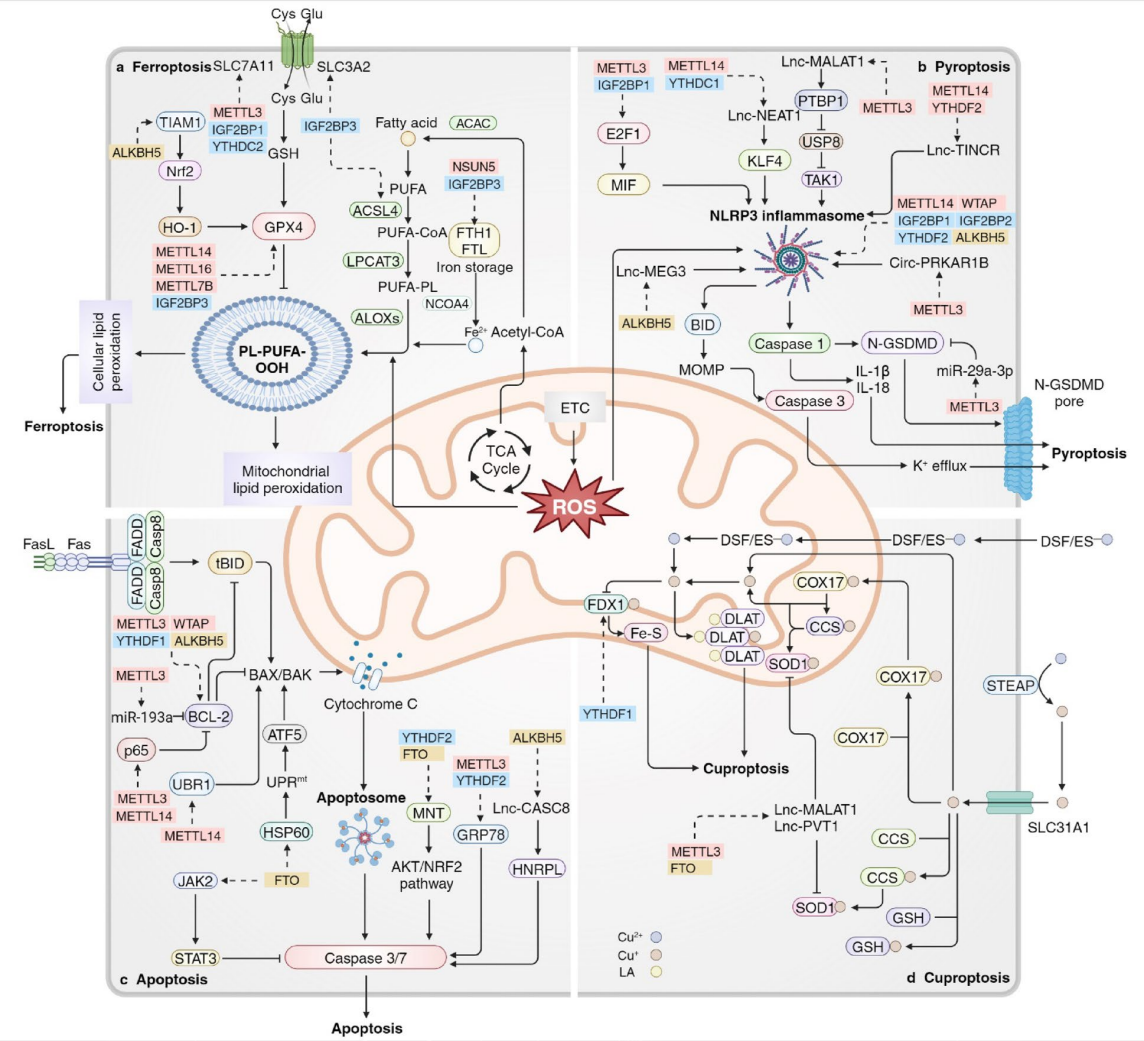
RNA modifications in mitochondria-related cell death. **a** Acetyl-CoA generated from the TCA cycle in the mitochondrial matrix is converted into PUFA-PL. Together with the ROS generated throughout the ETC on the IMM and Fe^2+^, PUFA-PLs are prone to oxidize into PL-PUFA-OOH that leads to cellular and mitochondrial lipid peroxidation, thus inducing ferroptosis. The GPX4 pathway acts as an anti-ferroptotic mediator. **b** The NLRP3 inflammasome activated by ROS induces caspase 1 activation and leads to the cleavage of IL-1β, IL-18 and GSDMD. N-GSDMDs form GSDMD pores that permeabilize the plasma membrane, leading to pyroptosis. **c** The intrinsic stimulus activates the BAX/BAK protein and induces MOMP, which leads to leakage of cytochrome C from the IMM. Cytochrome C activates the apoptosome, followed by caspase 3/7 activation, leading to apoptotic cell death. **d** Mitochondrial Cu(I), either from Cu(II) transported by DSF/ES or through SLC31A1, binds to FDX1 and lipoyled DLAT, destabilizing the Fe-S complex in IMS as well as DLAT oligomerization, leading to cuproptosis. The RNA modifications modifiers that regulate the key molecules involved in mitochondria-related cell death include writers in red, readers in blue and erasers in yellow. PUFA-PL polyunsaturated fatty acid-containing phospholipids, IMM inner mitochondrial membrane, IMS mitochondrial intermembrane space, GPX4 glutathione peroxidase, MOMP mitochondrial outer membrane permeabilization, DSF disulfiram, ES elesclomol, FDX1 ferredoxin 1, DLAT dihydrolipoyl transacetylase, LA lipoic acid

### RNA modifications in mitochondria-related ferroptosis

Ferroptosis is an iron-dependent form of regulated cell death that is initiated by excessive lipid peroxidation and is implicated in multiple diseases [210, 211]. The production of ROS in the ETC, as well as the release of Fe^2+^ from the ferritin components (FTH1 and FTL), can lead to the oxidation of polyunsaturated fatty acid-containing phospholipids (PUFA-PLs) derived from acetyl-CoA in the TCA cycle within mitochondria. This oxidation results in the formation of PL-PUFA-OOH, which promotes lipid peroxidation on mitochondrial, endoplasmic reticulum and cellular membranes, ultimately leading to cell ferroptosis [210]. Glutathione (GSH), synthesized from Cys imported through the system Xc^−^ constructed by SLC7A11 and SLC3A2, serves as a reducing cofactor to enhance glutathione peroxidase (GPX4) activity, thereby reducing lipid hydroperoxides to lipid alcohols, which forms an effective antioxidation system that protects cells from ferroptosis [210].

RNA modifications are closely correlated with and play a vital role in the regulation of mitochondria-related ferroptosis (Fig. 4a). In glioblastoma and hepatocarcinoma, the expression level of SLC7A11 can be upregulated in an m^6^A-dependent manner with the assistance of METTL3 and IGF2BP1 via the inhibition of SLC7A11 mRNA degradation and increased SLC7A11 mRNA splicing and maturation [212, 213]. However, contradictory roles of RNA modifications in SLC7A11 mRNA have been identified in alternative disease models. Ma L et al. demonstrated that YTHDC2-mediated m^6^A modification could destabilize SLC7A11 mRNA, and thereby promoting its decay in PDX mouse models of lung adenocarcinoma. Additionally, Li N et al. reported a negative correlation between the expression level of METTL3 and SLC7A11 in aortic dissection, suggesting that m^6^A modification of SLC7A11 mRNA may reduce its expression and facilitate ferroptosis [214, 215]. Moreover, ALKBH3-mediated m^1^A demethylation suppresses ferroptosis in KG-1 cells by increasing ATF4 expression and following increased SLC7A11 expression, thereby promoting the development of AML [216]. Further investigation and examination are required to elucidate the precise functions of RNA modifications in SLC7A11 mRNA across various disorders and under diverse conditions and environmental factors. Another member of the system Xc^−^, SLC3A2, can also be positively regulated by METTL3 and IGF2BP3 in lung adenocarcinoma cells [217].

METTL14, METTL16, METTL7B and IGF2BP3 jointly and positively mediate GPX4 mRNA stability and protein levels, suppressing ferroptosis in lung carcinoma, breast cancer and osteoclasts [217–220]. Moreover, NSUN2 modulates GPX4 mRNA stability via m^5^C methylation, inhibiting hepatocyte ferroptosis [221]. ALKBH5 can downregulate GPX4 via demodifications of TIAM Rac1 associated GEF 1 (TIAM1) mRNA, which leads to reduced TIAM1 expression. This, in turn, downregulates the Nrf2/HO-1 axis, resulting in advanced ferroptosis and repressed cancer cell proliferation [222, 223]. NSUN5 methylates FTH1/FTL mRNA via m^5^C modification by targeting the 5′UTR/3′UTR, inducing the degradation of FTH1/FTL mRNA, and leading to decreased iron storage levels and the accumulation of free ferrous ions, causing ferroptosis [42]. Xu X, et al. presented an alternative perspective, suggesting that IGF2BP3 has the ability to interpret the m^6^A signal in FTL1/FTL mRNA and sustain its stability, leading to an elevation in FTH1/FTL levels, therefore inhibiting ferroptosis and enhancing tumor development [217].

The RNA modifications on SLC7A11/GPX4 axis and FTH1/FTL axis significantly moderate the formation and activity of PL-PUFA-OOH, which promotes mitochondrial lipid peroxidation, leading to cell ferroptosis.

### RNA modifications in mitochondria-related pyroptosis

Pyroptosis is characterized by cell swelling, membrane perforation, and inflammatory responses, representing a lytic and inflammatory form of regulated cell death. This process is canonically initiated by inflammasomes and the caspase family, and is executed by gasdermin family proteins [224]. ROS induce the activation of the NOD-, LRR- and pyrin domain-containing protein 3 (NLRP3) inflammasome complex, which in turn activates inflammatory caspases, including caspase 1/4/5/11. These caspases cleave the pro-inflammatory cytokines IL-1β and IL-18, as well as gasdermin D (GSDMD), facilitating their maturation. The activated GSDMD then forms N-GSDMD pores that permeabilize the plasma membrane, ultimately resulting in pyroptotic cell death [5, 225–228].

RNA modifications play a crucial role in regulating mitochondria-associated pyroptosis at the post-transcriptional level (Fig. 4b). NLRP3 mRNA can be stabilized by METTL3, METTL14, WTAP, IGF2BP1 and IGF2BP2, thereby enhancing the NLRP3/caspase-1/GSDMD-related classical pyroptosis signaling pathway in contexts such as kidney injury in diabetic nephropathy, arsenic-induced hepatic insulin resistance, human umbilical cord mesenchymal stem cells and gefitinib-acquired resistance in lung cancer [229–232]. In contrast, Xiao J et al. reported a divergent finding, indicating that the ALKBH5-YTHDF2 m^6^A modification axis may inhibit NLRP3, thereby mitigating the progression of rheumatoid arthritis progression [233]. Moreover, FTO binds with NLRP3 and inhibits its expression, leading to suppressed pyroptosis and alleviated diabetic kidney injury [234]. Furthermore, IGF2BP1 can identify a METTL3-induced m^6^A recognition site within the 3’-UTR of E2F transcription factor 1 (E2F1) mRNA, which enhances E2F1 mRNA stability. E2F1 serves as a transcription factor that promotes the expression of macrophage migration inhibitory factor (MIF), which is a subunit of the NLRP3 inflammasome [235]. Nonetheless, RNA modifications of different target mRNAs exhibit contrasting effects on pyroptosis. YTHDF1 can positively regulated the expression of WW domain containing E3 ubiquitin protein ligase 1 (WWP1) expression through RNA modifications, which increases the ubiquitination of NLRP3 and subsequetly decreases NLRP3 expression levels, thereby alleviating sepsis [236, 237].

Moreover, Yang Q et al. demonstrated that lncRNA nuclear paraspeckle assembly transcript 1 (NEAT1) is positively regulated by METTL14 and YTHDC1, which further advances the transcriptional activation of NLRP3 through its interaction with Kruppel-like factor 4 (KLF4), resulting in ameliorated atherosclerosis [33]. Another lncRNA, metastasis associated lung adenocarcinoma transcript 1 (MALAT1), can also be upregulated by METTL3, promoting ubiquitin specific peptidase 8 (USP8) mRNA degradation via interaction with polypyrimidine tract binding protein 1 (PTBP1). This interaction positively modulates the ubiquitination and protein stability of nuclear receptor subfamily 2 group C member 2 (NR2C2), inducing pyroptosis and inflammation in M1 macrophages and aggravating liver fibrosis [238]. The lncRNA TINCR is subject to downregulation and accelerated degradation by METTL14 and YTHDF2, resulting in decreased mRNA stability of NLRP3 in diabetic cardiomyopathy [239]. In the context of cerebral ischemic stroke, reduced level of FTO positively modulates the stability of the lncRNA maternally expressed gene 3 (MEG3), thereby activating neuronal pyroptosis through the NLRP3/caspase-1/GSDMD signaling cascade [240].

RNA modifications not only modulate mitochondrial ROS production through the OXPHOS, but also regulate the expression and stability of key components within the downstream pyroptosis pathway. This establishes a critical regulatory axis where RNA modifications intricately control mitochondria-involved pyroptosis by influencing both the upstream mitochondrial ROS signal and the downstream cell death machinery. Therefore, elucidating this precise regulatory interplay is essential for a comprehensive understanding of cell death in disease pathogenesis and for providing novel targets for therapeutic intervention.

### RNA modifications in mitochondria-related apoptosis

Apoptosis, a key form of regulated cell death, plays crucial roles in various biological processes [241]. The activation of the effector pro-apoptotic B cell lymphoma 2 (BCL-2) proteins BAX and BAK result in mitochondrial outer membrane permeabilization (MOMP), which facilitates the release of cytochrome c from the IMS into the cytosol. Once released, cytochrome c binds to apoptotic peptidase activating factor 1 (APAF1) to form an apoptosome, which, in conjunction with extrinsic signals, further cleaves and activates caspase 3 and 7, ultimately leading to apoptosis. Additionally, caspase 8 can activate tBID and BAX/BAK, connecting the extrinsic and intrinsic apoptotic pathways [5]. Importantly, the anti-apoptotic BCL-2 proteins, particularly BCL-2, play a pivotal role in inhibiting cell death by deactivating tBID, BAX/BAK and BH3-only proteins [5].

Importantly, RNA modifications play a crucial role in the regulation of mitochondria-related apoptosis at the epitranscriptomic level (Fig. 4c). Various investigations have identified conflicting roles of m^6^A modification of BCL-2 mRNA. Lin S et al. and He Y et al. reported that METTL3 positively modulates the stability of BCL-2 mRNA and inhibits apoptosis through YTHDF1-mediated m^6^A modifications in temporomandibular joint osteoarthritis and gastric cancer [242, 243]. Conversely, another two studies conducted by Wang Y et al. and Zhu H et al. revealed that ALKBH5 enhances the stability of BCL-2 mRNA, thereby suppressing apoptosis in epithelial ovarian cancer, while the overexpression of WTAP can lead to the downregulation of BCL-2 in an m^6^A-dependent manner in breast cancer [244, 245]. m^6^A-marked transcripts are actively targeted for degradation through direct binding to YTHDF2, which recruits the CCR4–NOT deadenylase complex, a complex responsible for mRNA decay, explaining the pro-stabilizing role of ALKBH5 on BCL-2 mRNA [246]. Furthermore, BCL-2 can also be indirectly downregulated through RNA modifications. Sepsis-induced overexpression of METTL3 promotes the maturation of miR-193a, which binds to the 3’UTR of BCL2L2, resulting in the downregulation of BCL2L2 [247]. Studies have demonstrated that in human umbilical vein endothelial cells, METTL3 and METTL14 can upregulate p65 expression, leading to decreased levels of BCL-2 and exacerbated atherosclerosis [248].

Moreover, the BAX/BAK protein can also be regulated through m^6^A modification. In spinal cord injury, the expression of the ubiquitin protein ligase E3 component N-recognin 1 (UBR1) is diminished due to METTL14-mediated RNA modifications, which subsequently increases the levels of BAX/BAK protein [249]. FTO mitigates adipocyte apoptosis by decreasing the m^6^A modification of HSP60 mRNA, leading to the suppression of the mitochondrial unfolded protein response (UPR^mt^) and the inactivation of the PKR/eIF2α/ATF5 axis, ultimately resulting in the downregulation of BAX/BAK [250].

Furthermore, the depletion of METTL3 or the knockdown of YTHDF2 enhances the mRNA stability of GRP78, resulting in the upregulation of caspase-3 expression [251]. In esophageal squamous cell carcinoma, the upregulation of the lncRNA cancer susceptibility candidate 8 (CASC8) through ALKBH5-mediated m^6^A demodifications stabilizes the CASC8 transcript, thereby negatively modulating the caspase-3 apoptotic pathway [252]. FTO and YTHDF2 regulate the expression of MAX network transcriptional repressor (MNT) via m^6^A modification, which mitigates cadmium-induced apoptosis through the activation of the AKT/NRF2 signaling pathway [253].

### RNA modifications in mitochondria-related cuproptosis

Cuproptosis, a novel form of regulated cell death that is dependent on copper metabolism, has been implicated in various diseases [254–256]. Cu(I) binds to ferredoxin 1 (FDX1), resulting in decreased stability of the Fe-S complex in IMS, which subsequently induces increased proteotoxic stress and ultimately leads to cuproptosis. The released free copper ions in the mitochondria can also be directly transferred to superoxide dismutase 1 (SOD1), which plays a crucial role in catalyzing the conversion of superoxide radicals into H_2_O_2_, thereby maintaining intracellular ROS homeostasis and preventing cell death [257, 258].

Several publications have provided a novel perspective on the role of RNA modifications in the regulation of mitochondria-related cuproptosis (Fig. 4d). Bioinformatics analyses have indicated that METTL3 and FTO could upregulate the expression of LncRNA-MALAT1 and LncRNA-PVT1, thereby positively mediating SOD1 activity and reducing ROS accumulation; however, the direct correlation between RNA modifications and cuproptosis has not been investigated [259]. Studies have demonstrated that c-MYC can upregulate FDX1 via YTHDF1 with increased sensitivity to cuproptosis in glioma cells and that copper stress promotes METTL16 lactylation, leading to elevated METTL16 expression, which positively mediates cuproptosis through the m^6^A modification of FDX1 mRNA. These findings suggest potential mechanisms for the posttranscriptional regulation of cuproptosis [16, 260].

### RNA modifications in mitochondria-related necroptosis

Necroptosis is a pro-inflammatory mode of cell death characterized by the release of damage-associated molecular patterns (DAMPs), which is initiated by TNF-α treatment [5, 261]. The formation of the necrosome followed by the phosphorylation, activation and translocation of mixed-lineage kinase domain-like pseudokinase (MLKL) to the plasma membrane, results in membrane permeabilization and subsequent cell death. Additionally, the mitochondria-derived ROS can enhance the necrosome assembly.

Significantly, RNA modifications are involved in the regulation of mitochondria-related necroptosis. It has been demonstrated that the adenosine-to-inosine (A-to-I) base modification of the left-handed Z-nucleic acid sensor (ZBP1) by adenosine deaminase acting on RNA 1 (ADAR1), through the Z-nucleic-acid-binding Zα domain of ADAR1, can inhibit ZBP1 activation and the subsequent MLKL-mediated necroptosis [262]. Further investigation is required to determine the involvement of RNA modifications in the regulation of mitochondria-associated necroptosis.

Regulated cell death plays dual roles across various diseases. In cancer treatment, the induction of regulated cell death is essential for suppressing tumor development and progression. Conversely, cell death should be inhibited in other diseases, including neurodegenerative diseases, viral infection and cardiovascular diseases, to avoid further tissue damage. Further work is warranted to explore the potential of RNA modifications in the regulation of mitochondria-related cell death as a means to target regulated cell death and diseases.

## RNA modifications in mitochondrial dynamics

**Fig. 5:**
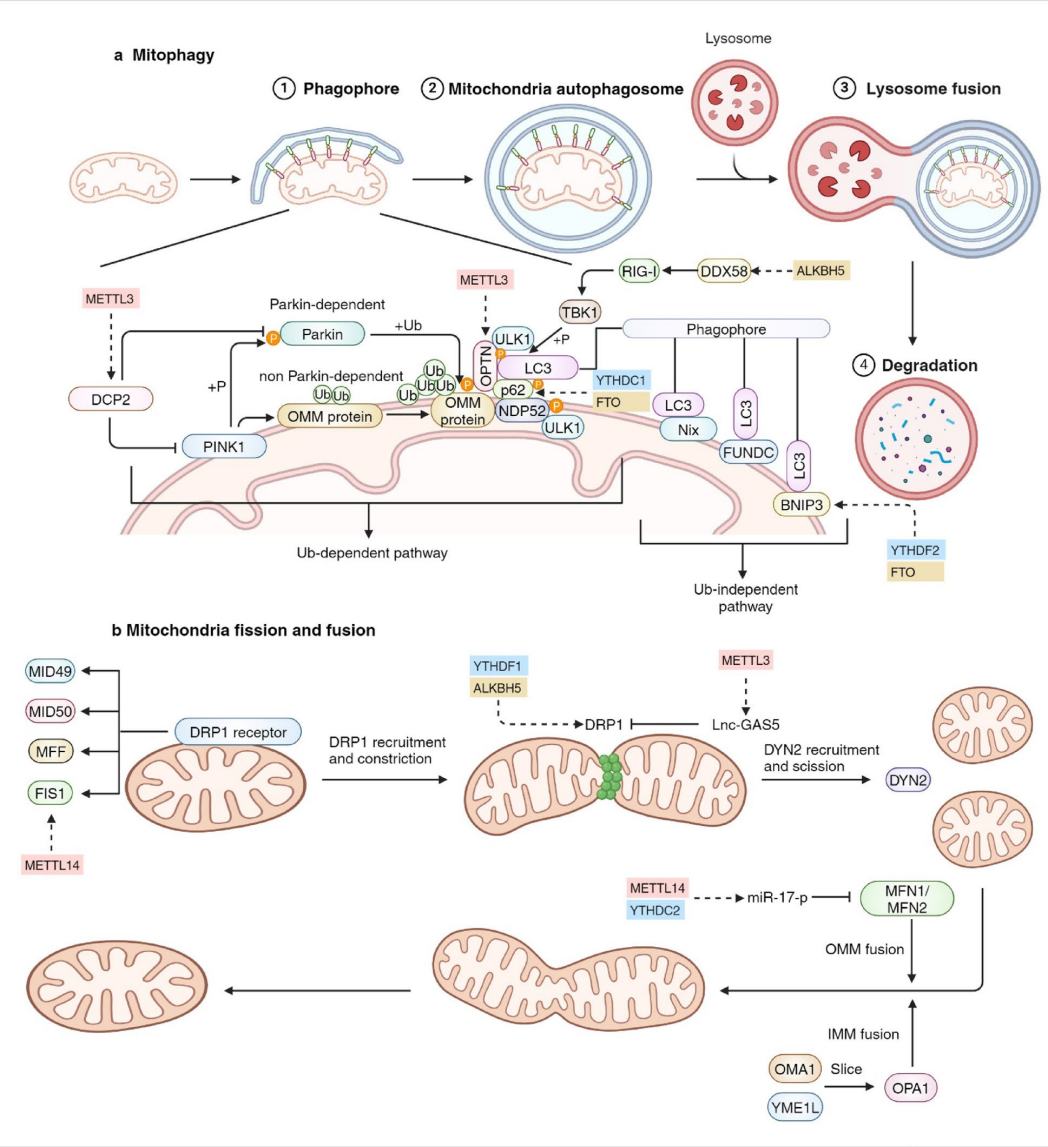
RNA modifications in mitochondrial dynamics. **a** Mitophagy is mainly composed of 4 processes: depolarization of mitochondria, formation of mitochondrial autophagosomes, fusion of mitochondrial autophagosomes and lysosomes, and degradation by lysosomes. The formation of mitochondrial autophagosomes are triggered by the PINK1-Parkin pathway and receptor-mediated pathway. **b** Mitochondrial fission is significantly mediated by DRP1. Mitochondrial fusion is triggered by OPA1 and MFN1/2. RNA modifiers that regulate the key molecules involved in mitochondrial dynamics include writers (red), readers (blue) and erasers (yellow). PINK1 PTEN-induced kinase 1, DRP1 GTPase enzyme dynamin-related protein 1, OPA1 optic atrophy protein 1, MFN 1/2 mitofusin 1/2

### RNA modifications in mitophagy

Mitophagy, a highly conserved cellular process that selectively eliminates dysfunctional or damaged mitochondria through autophagy, is pivotal for the regulation of mitochondrial quantity and quality control, thereby maintaining the homeostasis of mitochondria-related biological processes [8, 263]. Mitophagy encompasses four key processes: the depolarization of mitochondria, the formation of mitochondrial autophagosomes, the fusion of mitochondrial autophagosomes and lysosomes, and the subsequent degradation by lysosomes. The PTEN-induced kinase 1 (PINK1)-Parkin pathway and the receptor-mediated pathway play significant roles in the formation of selective mitochondrial autophagosomes.

RNA modifications play a crucial role in the mediation of mitophagy, which has been extensively reported in recent years (Fig. 5a). METTL3 induces the degradation of decapping protein 2 (DCP2), resulting in reduced inhibition of PINK1 and Parkin with enhanced mitophagy, alleviated tumor cell dysfunction levels and increased chemotherapy resistance in small cell lung cancer (SCLC) [79]. However, another study revealed an opposing effect of mitophagy on cancer cells, as the platelet-derived growth factor (PDGF)-METTL3 axis could downregulate the expression of optineurin (OPTN), inhibiting mitophagy and promoting cancer stem cell maintenance in glioblastoma [264, 265]. Moreover, the expression of sequestosome-1 (p62/SQSTM1) can be positively modulated by YTHDC1 and FTO, leading to the upregulation of mitophagy [266, 267]. Furthermore, in head and neck squamous cell carcinoma, ALKBH5 and HNRNPC downregulate the RNA modification levels of DDX58 mRNA and translation of the RIG-I protein, which induces the downregulation of TBK1 and mitophagy [268]. In addition, PINK1 insufficiency sensitizes tumors to mitochondria-centric combination therapies, including Mdivi-1 and indomethacin, potentiating the translational opportunities of RNA modification-mediated regulation of PINK1 to cancer treatment [269].

Beyond the PINK1-Parkin pathway, the receptor-mediated pathway can also be modulated via RNA modifications. In breast cancer, FTO mediates m^6^A demodifications in the 3’UTR of BCL2 interacting protein 3 (BNIP3) mRNA and induces its degradation, promoting breast cancer cell proliferation [270]. Nevertheless, Li T et al. and Cai X et al. have independently determined that the m^6^A modification site of BNIP3 mRNA can be identified by the reader YTHDF2, leading to increased BNIP3 mRNA degradation, which inhibits mitophagy and ameliorates myocardial ischemia-reperfusion injury (MIRI) [271, 272]. Therefore, it is imperative to further differentiate and elucidate whether RNA modifications exert a positive or negative effect on BNIP3 expression.

### RNA modifications in mitochondrial fission and fusion

Mitochondrial fission contributes to the mitochondrial quality control by facilitating the elimination of dysfunctional or damaged mitochondria, while mitochondrial fusion is conducive to merging and exchanging the intramitochondrial contents and helps to maintain mitochondrial function [8, 273]. Mitochondrial fission is significantly associated with the recruitment of the GTPase enzyme dynamin-related protein 1 (DRP1) through various OMM DRP1 receptor proteins, which leads to the constriction of the mitochondrial tubules and mitochondrial fission [274, 275]. Mitochondrial fusion is composed of the OMM fusion mediated by mitofusin (MFN) 1/2 and IMM fusion mediated by optic atrophy protein 1 (OPA1), resulting in the mixing of intramitochondrial components [8, 276].

RNA modifications play a significant role in the regulation of mitochondrial fission and fusion (Fig. 5b). DRP1 can be directly modulated by YTHDF1 and ALKBH5 and indirectly modulated by METTL3, which induces lncRNA GAS5 degradation in a YTHDF2-dependent manner. YTHDF2 interacts with and suppresses DRP1, thereby increasing cardiac fibroblast proliferation in cardiac fibrosis [277, 278]. Recently, Deng P et al. discovered that the lncRNA Gm10532 can enhance mitochondrial fission 1 (FIS1) expression and promote mitochondrial fission by recruiting METTL14 [279]. Furthermore, METTL14 inhibits the decay of pri-miR-17 mRNA by reducing the recognition of YTHDC2 at the “GGACC” binding site, resulting in decreased miR-17-5p levels and increased MFN2 expression [34]. Overall, there is currently limited study on the specific role of RNA modifications in mitochondrial dynamics. However, further investigations are highly valuable to uncover the intricate connections and regulatory processes involved.

## RNA modifications in the regulation of mitochondria-related antitumor immunity

**Fig. 6:**
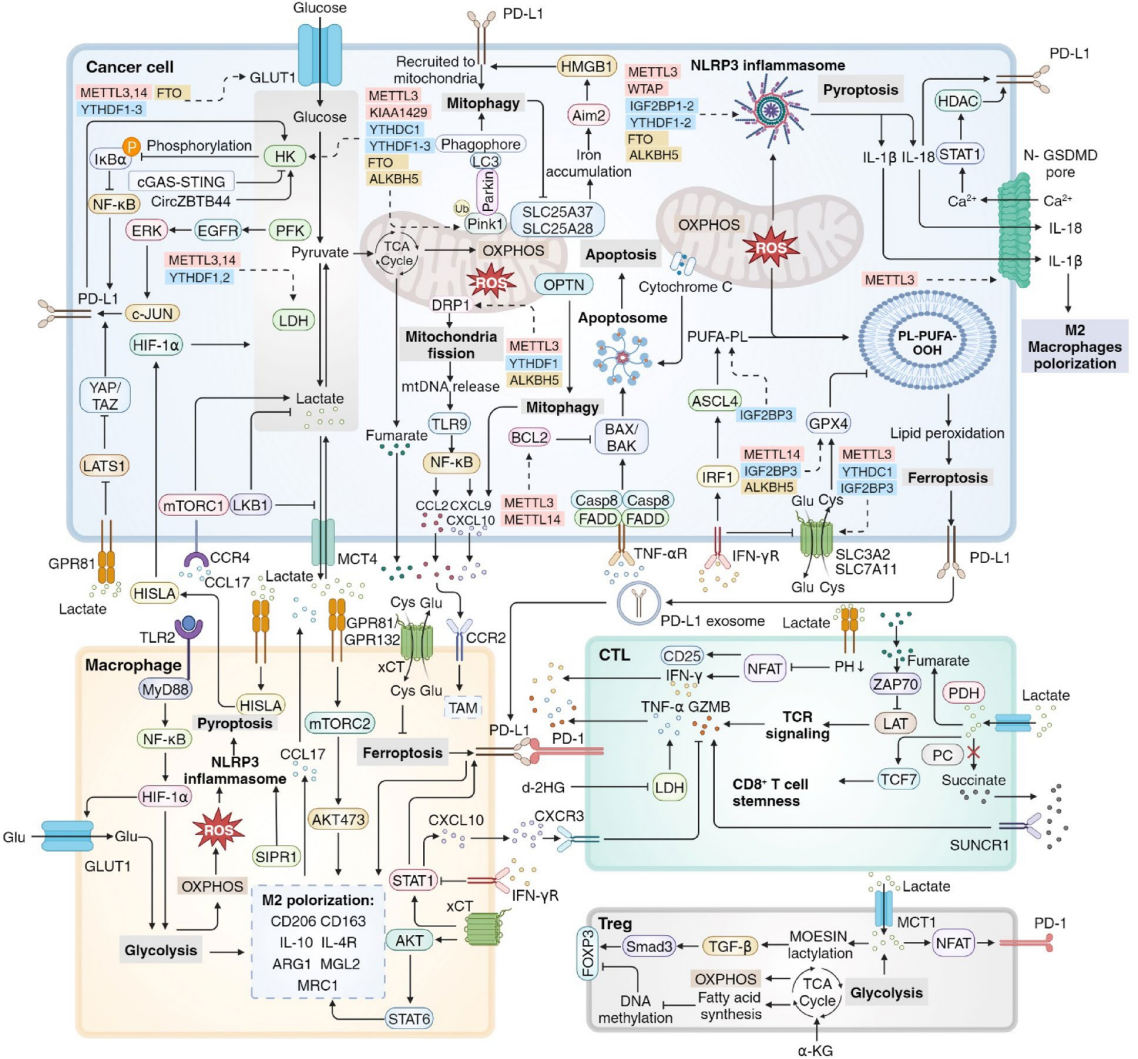
RNA modifications in the regulation of mitochondria-related tumor microenvironment. Mitochondria-related glucose metabolism, regulated cell death and mitochondrial dynamics are significantly correlated with the dynamic changes of the TME and tumor immune response. The role of RNA modifications in the regulation of the TME has not been discussed, yet it can be hypothesized and deduced. RNA modifiers that regulate the key molecules of mitochondria-related TME include writers (red), readers (blue) and erasers (yellow). TME tumor microenvironment

Here, we summarize the potential role of RNA modifications in regulating mitochondria-related antitumor immunity based on recent findings, with the aim of providing new scientific research directions for tumor treatment at the posttranscriptional level (Fig. 6).

### RNA modifications in mitochondria-related glucose metabolism in the regulation of antitumor immunity

#### RNA modifications in glycolysis in tumor cells

The intimate metabolic interplay between glucose metabolism in cancer cells and immune cells in the TME is crucial for cancer immunity. Pan-cancer single-cell RNA-seq data revealed that GLUT1 is highly expressed in cancer cells, while GLUT3 exhibits elevated expression in immune cells, indicating distinct regulatory mechanisms governing glucose transporter families in cancer and immune cells, with RNA modifications potentially serving as a regulatory factor [280]. A dual-targeting strategy combining GLUT1/NSUN2 axis inhibitor WZB117 with PD-L1 blockade, which synergistically suppressed tumor evolution and reversed immunosuppression in preclinical models, suggesting a novel synergistic therapeutic strategy for treatment-resistant HCC [41].

In addition to GLUTs, high glucose levels in glioblastoma and breast cancer cells, promote the dissociation of HK2 from mitochondria and positively mediate PD-L1 expression via the IκBα/NF-κB pathway [281, 282]. Conversely, it has also been reported that PD-L1 enhances glycolysis in NSCLC by upregulating HK2 [283]. An in vivo study has confirmed that the cGAS-STING pathway can inhibit HK2 to restrict tumor aerobic glycolysis and promote antitumor immunity [284]. Considering that RNA modifications significantly regulate the expression of HK2, it is reasonable to hypothesize that m^6^A modifiers, including METTL3, METTL14, KIAA1429, YTHDC1, YTHDF1-3, IGF2BP2, FTO and ALKBH5, may mediate PD-L1 expression and tumor immune escape through the regulation of HK2 mRNA modifications.

The role of another key enzyme in glycolysis, PFK, in antitumor immunity has been obscured by recent findings. PFKFB3 can upregulate PD-L1 expression via the EGFR/ERK/c-Jun pathway in renal cell carcinoma. Conversely, elevated PD-L1 expression can reciprocally enhance PFKFB3 levels [285]. However, a separate study indicated that the inhibition of PFKFB3 activated HIF-1α and transcriptionally upregulated PD-L1 expression with reduced levels of CD8 and GZMB and shorter survival times in ESCC patients, suggesting that PFKFB3 may act as a protumor-immunity factor [286]. RNA modifications mediated by METTL3, METTL14, IGF2BP2, YTHDF1-3 and FTO directly or indirectly regulate PFK expression, indicating that RNA modifications may modulate PD-L1 expression through PFK. Further investigation is needed to determine whether the distinct role of PFK in antitumor immunity is influenced by RNA modifications.

#### RNA modifications in the regulation of tumor cell-derived lactate

Lactate has been shown to play a pivotal role in tumor immunosuppression, with potential involvement of RNA modifications. Tumor cell-derived lactate accumulates in the TME and is sensed by its receptor, G protein-coupled receptor 81 (GPR81). The activation of GPR81 leads to the activation of the TAZ/TEAD pathway which transcriptionally induces PD-L1 expression [287]. The production and secretion of lactate can be modulated by the tumor suppressor gene serine/threonine kinase *STK11* encoding the LKB1 protein (STK11/LKB1). Notably, mutations in *STK11*/LKB1 are associated with increased expression of MCT4 and enhanced lactate secretion, as well as the polarization of M2 macrophages and the suppression of cytotoxic T cells [288]. An in vivo study demonstrated that tumor-derived lactate activated macrophage G protein-coupled receptor 132 (GPR132), promoting the tumor associated macrophage (TAM) phenotype in breast cancer [289]. A similar molecular mechanism was identified in lung cancer, where OLFR78 collaborates with GPR132 to mediate the lactate-induced generation of TAMs [290]. Moreover, lactate facilitates M2 polarization through the mTORC2 and ERK signaling pathway [291]. Interestingly, lactate-induced M2 TAMs enhance tumor cell proliferation in pituitary adenoma via the CCL17/CCR4/LDH/lactate axis. A feed-forward loop exists between TAMs and tumor cells, as lactate upregulates HIF-1α-stabilizing long noncoding RNA (HISLA) in macrophages, subsequently inhibiting the hydroxylation and degradation of HIF-1α in breast cancer [292].

Lactate in the TME can impede the activation and functionality of cytotoxic CD8^+^ T cells. Numerous studies have demonstrated various lactate-induced metabolic alterations in CD8^+^ T cells [293, 294]. In the absence of extracellular lactate, cytotoxic T cells depend on pyruvate carboxylase (PC) to replenish TCA cycle intermediates and shunt succinate out of the TCA cycle to facilitate autocrine signaling via the succinate receptor (SUCNR1), ultimately promoting the production and secretion of cytotoxic cytokines and GZMB. However, extracellular tumor-derived lactate reduces PC-mediated anaplerosis and redirects pyruvate flux from PC to PDH, reactivating succinate dehydrogenase (SDHA) and resulting in succinate oxidation rather than secretion. This sequence of events leads to increased production of fumarate but a decreased capacity to activate SUCNR1 due to reduced succinate secretion, which impairs CD8^+^ T cell effector function and inhibits antitumor immunity. Furthermore, recent studies have examined the positive role of lactate in enhancing the stemness of CD8^+^ T cells, which appears slightly contradictory to findings regarding its immunosuppressive role reported in other studies. Recent studies have confirmed that the administration of lactate inhibits the activity of histone deacetylase, leading to increased acetylation at H3K27 of the TCF7 super enhancer locus and increased TCF1/TCF7 gene expression, thereby upregulating CD8^+^ T cell stemness [295]. Conversely, Hermans D et al. reported that LDH inhibition rewired IL-2-induced effector-like metabolism, promoted pyruvate entry into the TCA cycle and subsequent OXPHOS, and suppressed the expression of IL-21-induced exhaustion markers, enhancing the formation of stem cell memory T cells (T_SCM_) and augmenting antitumor immune responses [296]. Therefore, the role of lactate in contributing to CD8^+^ T cell stemness warrants further investigation.

Furthermore, lactate in the TME plays a critical role in the functionality of regulatory T (Treg) cells [297]. Treg cells actively uptake lactate through MCT1, which facilitates the NFAT1 translocation into the nucleus, thereby promoting the expression of PD-1 and contributing to tumor immune inhibition [298]. The lactylation of lysine residues in MOESIN improves its interaction, subsequently activating the TGF-βRI/SMAD3/FOXP3 signaling cascade and augmenting Treg functions [299]. Research indicates that lactate derived from tumors has a negative effect on NK cells, diminishing NFAT levels and impairing NK cells activation, which results in reduced production of IFN-γ and inhibited tumor immunosurveillance [294]. In hepatocellular carcinoma, tumor-derived lactate induces the expression of PD-L1 on neutrophils via the MCT1/NF-κB/COX-2 signaling cascade, leading to decreased T cell cytotoxicity and compromised antitumor immunity [300].

Importantly, RNA modifications participate in the regulation of lactate production in tumor cells via the expression of LDH. m^6^A modifiers that regulate LDH expression, including METTL3, METTL14, YTHDF1 and YTHDF2, might play a pivotal role in the modulation of lactate production, secretion and accumulation in the TME.

#### RNA modifications in the regulation of the TCA cycle and OXPHOS in tumor cells

Fumarate, a metabolite generated in the TCA cycle, plays a significant role in tumor immunosurveillance. Depletion of Fumarate hydratase in tumor cells causes an accumulation of fumarate in the TME, which directly succinates ZAP70 at C96 and C102 and effectively blocks the phosphorylation of the ZAP70 substrate LAT as well as the downstream TCR signaling pathway, leading to suppressed CD8^+^ T cell activation and reduced IFN-γ, TNF-α and GZMB production [301]. Tumor cell metabolism also generates FFAs that promote fatty acid oxidation (FAO) in TAMs, which in conjunction with lactate produced by anaerobic glycolysis and the enhanced OXPHOS in macrophages, collectively induces the M2 polarization of these cells, leading to the formation of an immunosuppressive tumor microenvironment, further enabling tumor cells to evade immune surveillance and sustain proliferation [302]. The TCA cycle can be indirectly regulated by RNA modifications through NSUN2 and ALKBH5, while the ETC can be mediated by METTL3, METTL8, WTAP, NSUN2, NSUN3, IGF2BP1, IGF2BP3 and ALKBH3, suggesting that RNA modifications may regulate antitumor immunity via the TCA cycle and OXPHOS in tumor cells.

#### RNA modifications in glucose metabolism in immune cells

The interaction between RNA modifications and glucose metabolism in the antitumor immune response has not been extensively reported, until recently, an interesting study produced by Chen T et al. revealed that the m^5^C writer NSUN2 is a glucose sensor that directly binds to glucose, which posttranscriptionally stabilizes three prime repair exonuclease 2 (TREX2) mRNA and sustains its expression. TREX2 degrades cytosolic DNA, repressing cGAS-STING activation, leading to less apoptosis of tumor cells and downregulated CD8^+^ T cell infiltration in the TME, ultimately enhancing resistance to immunoblockade therapy [17].

In macrophages, GLUT1 and glycolysis can be upregulated through the tumor-derived exosome (TDE)/TLR2/NF-κB/HIF-1α signaling pathway, leading to increased cellular lactate, which subsequently feeds back on NF-κB to further enhances PD-L1 expression [303]. In CD8^+^ T cells treated with metformin, GLUT1 can be upregulated by mitochondrial ROS, leading to an increase in glycolysis and IFN-γ production. Additionally, mtROS trigger NF-E2-related factor 2 (NRF2) activation and mTORC1, while mTORC1, in turn, activates NRF2 in a p62-dependent manner, which enhances autophagy, glutaminolysis and the production of α-KG, thereby supporting CD8^+^ T cells proliferation [304]. Regarding ENO, ENO1-specific Tregs accumulate in the tumor tissue of pancreatic cancer, accompanied by decreased levels of ENO1-specific Th17 cells, highlighting a possible role in promoting pancreatic cancer progression [305]. Treg differentiation is associated with glucose metabolism, as increased α-KG facilitates the TCA cycle and OXPHOS in Tregs, thereby enhancing PUFA generation and triacylglyceride synthesis, which alters the DNA modifications profile of naive CD4 T cells, significantly reducing FOXP3^+^ Treg differentiation and increasing inflammatory cytokine production [306]. Further investigations are warrented to determine the role of RNA modifications in the regulation of glucose metabolism in immune cells in the TME.

### RNA modifications in mitochondria-related regulated cell death in the regulation of antitumor immunity

#### RNA modifications in ferroptosis in the regulation of antitumor immunity

Mitochondria-related ferroptosis is pivotal for the regulation of antitumor immunity. Inhibition of the anti-ferroptosis factor SLC7A11/SLC3A2 upregulates PD-L1 expression in tumor cells via IRF4/EGR1. PD-L1 in tumor cells can be secreted through exosomes into the TME, which leads to M2 macrophage polarization [307]. IFN-γ released from CD8^+^ T cells downregulates the expression of SLC7A11/SLC3A2 by activating the JAK/STAT pathway, which promotes tumor ferroptosis, contributing to the antitumor efficacy of immunotherapy [308, 309]. Analysis of the human transcriptome before and during nivolumab therapy further revealed that clinical benefits correlate with reduced Xc^−^ expression and increased IFN-γ [308]. In addition, IFN-γ secreted by CD8^+^ T cells stimulates ASCL4 expression in tumor cells through IRF1, which leads to the upregulation of ferroptosis in tumor cells, improving the immune checkpoint blockade (ICB)-induced antitumor immunity [310]. RNA modifiers, including METTL3, IGF2BP1, YTHDC2, which modulate the stability of SLC7A11 mRNA, and IGF2BP3, which mediates the expression of SLC3A2 and ACSL4, can be theoretically hypothesized to be correlated with the regulation of antitumor immunity, which deserves further investigation.

Mitochondria-related ferroptosis in immune cells is also a vital regulator of immunosurveillance. Recently, Tang B et al. discovered that Xc^−^ in macrophages can induce M2 macrophage phenotype shifting via the SOCS3-STAT6-PPAR-γ signaling pathway [311]. Moreover, the ferroptosis in TAMs significantly increases PD-L1 expression in macrophages and, interestingly improves the efficacy of anti-PD-L1 therapy [311]. Furthermore, Xc^−^ in macrophages can activate the JAK/STAT1 signaling cascade and induce the expression and secretion of PD-L1 and CXCL10, while it can also activate the AKT/STAT6 signaling pathway and upregulate M2 polarization [312]. Although the RNA modifications of ferroptosis-related genes mRNA in immune cells have not been reported, it is predictable that they might participate in the regulation of antitumor immunity.

#### RNA modifications in apoptosis in the regulation of antitumor immunity

Another type of regulated cell death, apoptosis, plays a significant role in the antitumor immune response. CD8^+^ T cells can induce apoptotic signals in tumor cells by activating caspase-3 and enhancing the T cell-dependent immune response [313]. BCL-2^+^ CD4^+^ T cells are enriched in Tregs with high expression of IL-10 and TGF-β, while BCL-2^+^ CD8^+^ T cells are associated with exhausted cells, reduced cytotoxicity and weak expression of GZMB and perforin [314]. In addition, genome-wide CRISPR screen analysis revealed that inhibition of BCL-2 enhanced dendritic cell (DC) antigen presentation as well as the capacity of DCs to control tumors and to synergize with PD-1 blockade [315]. RNA modifications modifiers that target BCL-2 mRNA, such as METTL3, WTAP, YTHDF1 and ALKBH5, may be correlated with the antitumor immune response via the regulation of apoptosis.

#### RNA modifications in pyroptosis in the regulation of antitumor immunity

Pyroptosis is commonly used as a strategy for cancer elimination. However, pyroptosis plays a different role in the antitumor immune response. The NLRP3 inflammasome in the tumor cells leads to markedly elevated IL-18 levels, which positively regulate PD-L1 expression and reduce the proportion of cytotoxic T cells [316]. In addition, it has been shown that NLRP3-mediated IL-18 receptor signaling acts as a stimulator of intratumoral T cell exhaustion, by activating the IL-2/STAT5 and AKT/mTORC1 signaling pathways [317]. Nevertheless, it seems that NLRP3/IL-18 are not always immunosuppressive factors, particularly in the context of colorectal cancer metastasis in the liver, where the NLRP3 inflammasome generated in Kuppfer cells induces immunosurveillance via NK cells but not CD8^+^ T cells, and IL-18 positively mediates NK cell maturation and tumoricidal activity independent of IFN-γ and suppresses metastasis [318]. IL-1β, another cytokine activated in pyroptosis, has also been shown to promote immune suppression through the establishment of an immunosuppressive milieu mediated by M2 macrophages and myeloid-derived suppressor cells (MDSCs) [319]. Inhibition of IL-1β significantly enhances the antitumor activity of anti-PD-1 therapy, accompanied by increased infiltration of CD8^+^ T cells [319]. The mechanism by which IL-1β induces M2 polarization was further investigated by Weichand B et al., who reported that IL-1β enhances TAMs formation via S1P receptor 1 (S1PR1) [320]. Remarkably, IL-1β plays a dual role. As mtDNA and mtROS induce IL-1β production in macrophages, IL-1β signals in DCs, leading to increased glycolysis, pro-inflammatory cytokines (IL-12, IFN-I) production as well as surface expression of T-cell stimulatory molecules (MHC, CD80,86) [321]. These advance the cross-priming activity of DCs by inducing mitochondrial Ca^2+^ signaling, elevating ROS production and increased IL-12, promoting radiation-induced antitumor immunity and helping overcome the radioresistance of tumors [322]. RNA modifications modifiers, including METTL3, WTAP, IGF2BP1-2, YTHDF1-2, FTO and ALKBH5, might play pivotal roles in antitumor immunity via the regulation of the NLRP3 inflammasomes, integrating the epitransciptional regulation and mitochondrial programs including ROS production, mtDNA sensing and metabolic reprogramming.

GSDMD has emerged as an effective target for tumor immunotherapy. GSDMD impairs cGAS-STING activation via K^+^ efflux, reducing IFN-γ production, and promotes PD-L1 expression via the import of Ca^2+^ followed by the activation of the STAT1 signaling pathway, alleviating the antitumor immune response [323]. Interestingly, GSDMD in cytotoxic T cells plays a distinct role, as the colocalization of GSDMD with GZMB has been reported to occur in the proximity of immune synapses, and GSDMD cleavage increases in activated CD8^+^ T cells, indicating that GSDMD is required for an optimal cytotoxic T cells response [324]. This duality underscores the nuanced role of RNA modifications in pyroptosis regulation in different TME components. While the impact of RNA modifications on GSDMD mRNA, which is modulated by METTL3, in the TME has not yet been uncovered, it is plausible to speculate that RNA modifications could potentially contribute to the antitumor immunity via regulating GSDMD.

### RNA modifications in mitochondrial dynamics in the regulation of antitumor immunity

#### RNA modifications of mitophagy in the regulation of antitumor immunity

Mitophagy is closely correlated with tumor immunosurveillance. PINK1-Parkin-mediated mitophagy modulates the degradation of SLC25A37 and SLC25A28, which are crucial for mitochondrial iron transport. SLC25A37 and SLC25A28 increase mitochondrial iron accumulation, leading to the upregulation of PD-L1 through the HIF-1α/AIM2/high mobility group box 1 (HMGB1) axis, resulting in immune dysfunction [325]. PINK1-mediated mitophagy can also recruit PD-L1 to mitochondria for degradation [326]. Moreover, the deficiency of Parkin is linked with PTEN degradation and enhanced AKT signaling, which leads to the dysregulation of antigen presentation and promotes tumor immune evasion [327]. Furthermore, in T_SCM_ PINK1-mediated mitophagy triggers cytosolic release of the mitochondrial phosphatase PGAM5 that dephosphorylates β-catenin, which drives Wnt signaling and compensatory mitochondrial biogenesis, inducing T_SCM_ formation and an improved immune response [328]. In addition, MEK inhibition facilitates OPTN-mediated mitophagy in lung cancer cells, leading to increased mt-DNA accumulation in the cytosol and the TLR9 signaling cascade, enhancing CD8^+^ T cells recruitment [329]. RNA modifications that directly or indirectly mediate the level of PINK1-Parkin-mediated mitophagy, is potentially correlated with antitumor immunity via RNA modifications modifiers.

#### RNA modifications in mitochondrial fission and fusion in the regulation of antitumor immunity

In recent years, mitochondrial fission and fusion have been reported to be closely related to antitumor immunity. In cancer cells, DRP1-mediated mitochondrial fission induces cytosolic mtDNA stress to enhance the CCL2 secretion from cancer cells via the TLR9-mediated NF-κB signaling pathway, which promotes M2 polarization [330]. Interestingly, in effector T cells however, DRP1 promotes correct thymocyte maturation by promoting T cell metabolic reprogramming and expansion, which allows efficient T cell extravasation from the blood and infiltration into tumors [331]. RNA modifications modifiers that directly or indirectly modulate the expression of DRP1, are believed to have a strong connection with the antitumor immune response via the regulation of DRP1-mediated mitochondrial fission. Investigations into whether the distinct roles of DRP1 and DRP1-dependent mitochondrial fission in different cells mentioned above are related to the different levels of RNA modifications that regulate DRP1 expression, are still needed.

## Discussion

In the proceeding context, we provided a concise overview of the latest findings about the involvement of RNA modifications in mitochondrial functions. However, many problems remain to be solved in this area, and more research needs to be devoted.

The connection between OXPHOS and RNA modifications was identified by hallmark findings that mitochondrial tRNA can be positively modulated via m^5^C modification, leading to the upregulated translation efficacy of OXPHOS complexes and increased tumor metastasis. It is reasonable to explore whether RNA modifications on mt-tRNAs regulate the regulated cell death in addition to glucose metabolism, for the ROS generated from OXPHOS are confirmed to induce various forms of cell death. In addition, whether RNA modifications can regulate mitochondrial mRNAs and other mitochondrial non-coding RNAs needs further investigation.

While existing literature has primarily concentrated on the role of RNA modifications in the biosynthesis of mitochondrial complexes, it is also known that proteins such as COA1 and COX18 are crucial for the subsequent complex assembly and membrane insertion. Nevertheless, the regulatory role of these RNA modifications in these processes remains uncharacterized. The elucidation of this regulatory mechanism would be pivotal to advancing our understanding of mitochondrial respiratory function in disease pathogenesis and tumor immune evasion, thereby identifying novel targets for therapeutic intervention. Consequently, a comprehensive investigation into this regulatory axis is warranted.

Emerging as a novel form of metal-ion-related cell death, the molecular mechanism of cuproptosis is currently being explored, and its correlation with RNA modifications was unknown; however, until recently, concrete evidence has shown that the lactylation of METTL16 upregulated cuproptosis through m^6^A modification of FDX1 mRNA. Further research should be conducted to evaluate whether other cuprotosis-related genes that are regulated by RNA modifications are correlated with antitumor immunity.

The direct correlation between RNA modifications and mitochondria-related antitumor immunity was recently discovered for the first time, as the m^5^C writer NSUN2 directly binds to glucose and represses the cGAS-STING pathway, leading to decreased apoptosis of tumor cells and CD8^+^ T cells infiltration. Therefore, further studies on the mechanisms by which RNA modifications control antitumor immunity are warranted. Cancer cells always exhibit increased glycolysis and decreased OXPHOS, resulting in elevated lactate secretion and the induction of immunosuppressive TME. Whether the metabolic reprogramming in cancer cells is due to the switch of RNA modifications remains to be determined. The efficiency of OXPHOS is critically dependent on the proper assembly of mitochondrial complexes, significantly influence the immunometabolism and anti-tumor immune activity of immune cells. Thus, the regulatory role of RNA modifications in mitochondrial complex assembly, especially assembly insertases and chaperones, warrants further investigation.

The TME is generally associated with reduced cell death of cancer cells and immunosuppressive cells, and increased cell death of cytotoxic cells. Unlike other types of cell death, pyroptosis in cancer cells has contradictory effects, as IL-18 and IL-1β are produced during the process of pyroptosis, inducing M2 polarization and cytotoxic T cells exhaustion. Hence, the role of RNA modifications in the regulation of pyroptosis process needs to be explored. Moreover, histone lactylation-regulated METTL3 promotes ferroptosis via m^6^A modification of ACSL4. Therefore, whether the high lactate levels in the TME may lead to the ferroptosis in tumor cells or immune cells, or whether this occurs through the histone lactylation of m^6^A modifiers needs further investigation.

Novel small-molecule inhibitors of RNA modifications modifiers have emerged in recent years, but few clinical trials have explored their use in clinical practice. A phase I study (NCT05584111) evaluated the safety and tolerability of STC-15, a METTL3 inhibitor, in advanced malignancies. The lately results indicates that treatment with STC-15 is well tolerated across pharmacologically active dose range with encouraging signs of clinical activity [332]. TEAEs were mainly mild to moderate, manageable and reversible. The DCR is 66% with 3 confirmed PR ongoing in angiosarcoma, IO-refractory NSCLC and thymoma in 29 patients evaluable for response [333]. A phase Ib/2 clinical trial (NCT06975293) of STC-15 is in progress, assessing the clinical activity and antitumor activity of STC-15 in combination with toripalimab. Combined treatment of small-molecule inhibitors of RNA modifications oncogenic modifiers and existing therapies, such as chemotherapy, targeted therapy and immunotherapy, may possibly augment the therapeutic effect and improve patient outcomes. Furthermore, a phase I study (NCT06762925) on the efficacy and mechanism of METTL3 peptide inhibitors in enhancing anti-tumor immune response in patients with urological tumors is conducting.

Although RNA modifications hold great potential as therapeutic targets, challenges of RNA-modification-targeted therapeutics remain. Given the highly context- and temporal-dependent nature of RNA modification functions, therapeutic molecules targeting these proteins must be dosed within a strictly controlled time window. For example, *Mettl3* knockout in mice is embryonically lethal, whereas METTL3 is oncogenic in various cancers [334]. An optimal strategy involves coupling these agents with targeted delivery and controlled release to ensure specific inhibition at disease sites. In addition, developing specific inhibitors for RNA-modifying and binding proteins is challenging due to their shared catalytic domains. The selectivity of proteins like FTO and ALKBH comes not from their conserved catalytic domain, but from their unique substrate-binding domain [335]. Consequently, inhibitors that simply compete with the α-KG co-factor risk inhibiting all α-KG-dependent enzymes. To ensure specificity, inhibitors must be designed to perturb the substrate-binding domain.

Moreover, drug developers must recognize that many RNA modification effectors possess additional functions independent of RNA modifications. For instance, METTL3 and METTL14 play critical roles in transcription and translation regulation even without methylase activity. Studies confirming that *METTL3* knockout affects a broader range of lineages than its catalytic inhibitor highlight the importance of the inhibition modality [336]. Therefore, using PROTACs to degrade the protein is beneficial as it simultaneously ablates both enzymatic and non-enzymatic functions [337]. Furthermore, the dual roles of RNA modification effectors like FTO and ALKBH5 (which can act as both oncogenes and tumor suppressors) must be considered. Although FTO and ALKBH5 have oncogenic roles in the majority of cancers studied, they can also be tumor suppressors [338, 339]. In these complex scenarios, achieving site-specific m^6^A demethylation on discrete targets may provide a viable path to attenuate tumorigenesis with minimal side effects [334].

## Conclusions

**Fig. 7:**
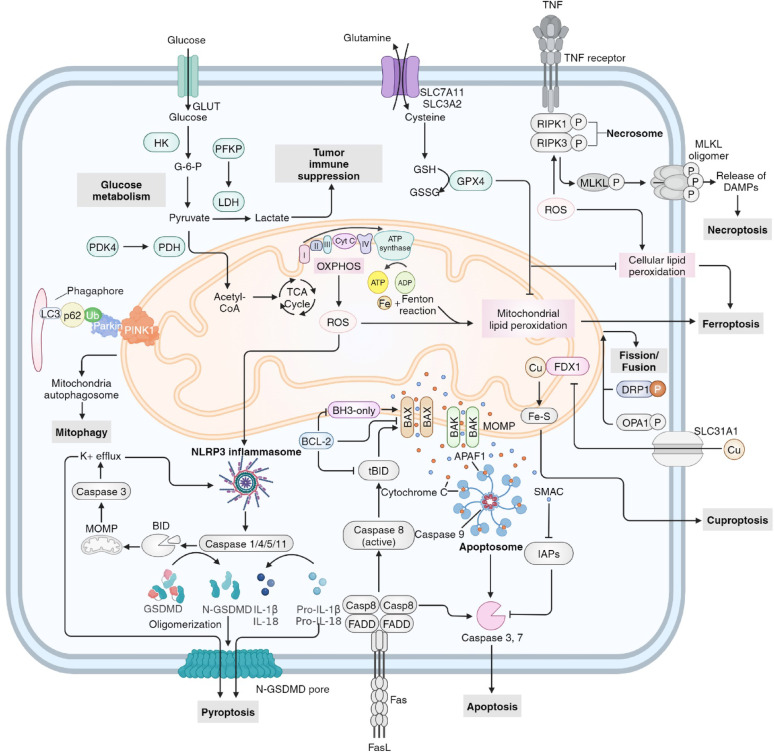
Overview of mitochondrial functions and their links with RNA modifications. Mitochondria play a crucial role in glucose metabolism, apoptosis, ferroptosis, necroptosis, pyroptosis, cuproptosis, mitophagy, mitochondrial fission and fusion and the tumor immune response. The key molecules involved in mitochondrial functions, which are reported to be associated with RNA modifications are depicted in color, whereas other molecules are in gray

In this review, we present the characteristics and role of RNA modifications in mitochondria-related glucose metabolism, regulated cell death and mitochondrial dynamics (Fig. 7), describe the molecular mechanisms underlying the posttranscriptional regulation of the related signaling pathways in different diseases (Table 4), and discuss the predictive role of RNA modifications in the mitochondria-related tumor microenvironment and tumor immune response, suggesting new therapeutic strategies for cancer and other diseases.

In conclusion, posttranscriptional regulation of mitochondria-related functions via RNA modifications is pivotal for disease development and treatment. With the advancement of research in this field, targeting mitochondrial functions through RNA modifications modifiers can be a strategy for various diseases.

**Table 4:** The relationship between mitochondria-related RNA modifications modifiers and diseases

Modifier	Type of RNA modification	Type of enzyme	Related mitochondrial functions	Changes of mitochondrial functions	Disease	Main target	Role	Ref
METTL3-METTL14, METTL16	m^6^A	Writer	Glycolysis	Upregulated	Liver cancer	LINCAROD, HIF-1α, WWP2	Promote	[174, 340, 341]
				Upregulated	Prostate cancer	CircRBM33	Promote	[194]
				Downregulated	Glioma	MiR-27b-3p	Suppress	[342]
				Upregulated	Esophageal squamous cell carcinoma	GLS2, APC	Promote	[343, 344]
				Upregulated	Lung cancer	ENO1, ABHD11-AS1, DLGAP1-AS2	Promote	[29, 187, 345]
				Upregulated	Gastric cancer	NDUFA4, HDGF, LHPP	Promote	[173, 198, 346]
				Upregulated	Breast cancer	LATS1	Promote	[161]
				Upregulated	Cervical cancer	HK2	Promote	[177]
				Upregulated	Colorectal cancer	LDHA, circQSOX1, PNN, COAD, SOGA1	Promote	[32, 167, 185, 347]
			OXPHOS	Upregulated	Gastric cancer	AVEN, DAZAP2, DNAJB1	Promote	[348]
			Ferroptosis	Downregulated	Breast cancer	GPX4	Promote	[219]
				Downregulated	Glioblastoma Hepatoblastoma	SLC7A11	Promote	[212]
				Downregulated	Aortic Dissection	GPX4, SLC7A11	Suppress	[215]
			Pyroptosis	Upregulated	Liver fibrosis	MALAT1	promote	[238]
				Upregulated	MI/R injury.	MiR-143-3p	promote	[349]
				Upregulated	Arsenic-induced hepatic insulin resistance Intervertebral disc degeneration Lung cancer	NLRP3	promote	[229–231]
				Upregulated	Crohn’s colitis	CircPRKAR1B	promote	[350]
			Apoptosis	Downregulated	Lung cancer Breast cancer	BCL-2	Promote	[244]
				Downregulated	Temporomandibular joint osteoarthritis	BCL-2	Suppress	[242]
			Mitophagy	Downregulated	Glioblastoma	OPTN	Promote	[264]
				Upregulated	Small cell Lung cancer	DCP2	Promote	[79]
				Upregulated	Colorectal cancer	Pri-miR-17	Promote	[34]
			Fission/fusion	Upregulated	Cd neurotoxicity	FIS1	promote	[279]
				Upregulated	Cardiac fibrosis	Gas5	promote	[277]
WTAP	m^6^A	Writer	Glycolysis	Upregulated	Colorectal cancer	PDK4	Promote	[193]
				Upregulated	Breast cancer	ENO1	Promote	[188]
				Upregulated	Colorectal cancer	FOXP3	Promote	[195]
				Upregulated	Ovarian cancer	MiR-200	Promote	[38]
			OXPHOS	Upregulated	Multiple myeloma	NDUFS6	Promote	[199]
			Pyroptosis	Upregulated	Diabetic nephropathy	NLRP3	promote	[232]
			Apoptosis	Downregulated	Breast cancer	BCL-2	Promote	[244]
KIAA1429	m^6^A	Writer	Glycolysis	Upregulated	Oral squamous cell carcinoma	PGK1	Promote	[184]
				Upregulated	Colorectal cancer	HK2	Promote	[179]
				Upregulated	Ovarian cancer	ENO1	Promote	[189]
YTHDF1 to YTHDF3	m^6^A	Reader	Glycolysis	Upregulated	Cervical cancer	PDK4	Promote	[27]
				Upregulated	Liver cancer	PDK4, PFKL	Promote	[27, 30]
				Upregulated	Lung cancer	ENO1, HK2, LDHA, LDHB and SLC2A1	Promote	[351]
				Upregulated	Breast cancer	LATS1, PKM2	Promote	[161, 352]
			Ferroptosis	Upregulated	Colorectal cancer	ATF4	Promote	[353]
			Cuproptosis	Upregulated	Glioma	FDX1	Promote	[260]
YTHDC1 and YTHDC2	m^6^A	Reader	Glycolysis	Upregulated	Pancreatic cancer	MiR-30d	Promote	[171]
			Ferroptosis	Downregulated	Lung cancer	SLC7A11	Suppress	[214]
IGF2BP1 to IGF2BP3	m^6^A	Reader	Glycolysis	Upregulated	Liver cancer	PDK4, LINCAROD	Promote	[27, 341]
				Upregulated	Cervical cancer	PDK4, MYC	Promote	[27, 165]
				Upregulated	Gastric cancer	HDGF	Promote	[173]
				Upregulated	Colorectal cancer	CircQSOX1	Promote	[185]
				Upregulated	Oral squamous cell carcinoma	CircFOXK2	Promote	[28]
			OXPHOS	Upregulated	Lung cancer	COX6B2	Promote	[37]
			Ferroptosis	Upregulated	Lung cancer	GPX4	Promote	[217]
				Upregulated	Hepatoblastoma	SLC7A11	Promote	[213]
			Pyroptosis	Upregulated	Acute kidney injury	E2F1	Promote	[235]
hnRNPCL2	m^6^A	Reader	OXPHOS	Upregulated	Colorectal Cancer	miR-483, miR-877, miR-676	Promote	[207]
FTO	m^6^A	Eraser	Glycolysis	Upregulated	Acute myeloid leukemia	PFKP	Promote	[31]
				Downregulated	Papillary thyroid cancer	APOE	Suppress	[172]
				Downregulated	Lung cancer	MYC	Suppress	[338]
				Downregulated	Colorectal cancer	HK2	Suppress	[178]
				Upregulated	Endometriosis	ATG5	Promote	[190]
				Upregulated	Gastric cancer	PRKAA1	Promote	[339]
				Downregulated	Diabetic nephropathy	NPAS2	Suppress	[169]
			Pyroptosis	Upregulated	Cerebral Ischemic Stroke	MEG3	Promote	[240]
				Downregulated	Diabetic kidney injury	NLRP3	Suppress	[234]
			Fission/fusion	Upregulated	Gastric cancer	Caveolin-1	Promote	[354]
ALKBH5	m^6^A	Eraser	Glycolysis	Downregulated	Papillary thyroid cancer	CircNRIP1	Suppress	[186]
				Upregulated	Lung cancer	ENO1	Promote	[187]
				Downregulated	Colorectal cancer	HK2	Suppress	[178]
			Ferroptosis	Upregulated	Thyroid cancer	TIAM1	Suppress	[223]
			Pyroptosis	Downregulated	Rheumatoid arthritis	NLRP3	Suppress	[233]
			Apoptosis	Downregulated	Ovarian cancer	BCL-2	Promote	[245]
			Fission/fusion	Downregulated	Liver fibrosis	DRP1	Promote (effector T cells)/Suppress (cancer cells and fibrosis)	[278]
NSUN1 to NSUN7	m^5^C	Writer	Glycolysis	Upregulated	Bladder cancer, Liver cancer	PKM2	Promote	[43, 191]
				Upregulated	Liver cancer	GLUT1	Promote	[41]
				Upregulated	Liver cancer	c-Myc	Promote	[208]
			TCA	Upregulated	Gastric cancer	LncRNA NR_033928	Promote	[197]
				Downregulated	Viral infection	OGDH	Suppress	[15]
			OXPHOS	Upregulated	Oral cancer	34 in mt-tRNA^Met^	Promote	[14]
				Upregulated	Neurodevelopmental disorders	48, 49 and 50 in mt RNA	Promote	[201]
			Ferroptosis	Upregulated	-	GPX4	-	[221]
YBX1	m^5^C	Reader	Glycolysis	Upregulated	Lung cancer	PFKFB4	Promote	[182]
ALKBH3	m^1^A	Eraser	Glycolysis	Downregulated	Cervical cancer	ATP5D	Suppress	[200]
			Glycolysis	Upregulated	Age-related macular degeneration	HK2	Promote	[40]
			Ferroptosis	Upregulated	Acute myeloid leukemia	ATF4	Promote	[216]
TRMT61A	m^1^A	Writer	Glycolysis	Upregulated	Head and neck squamous cell carcinoma	tRNA	Promote	[166]
TRMT10C	m^1^A	Writer	mt tRNA	Upregulated	Alzheimer’s disease	tRNA	Suppress	[39]
METTL8	m^3^C	Writer	OXPHOS	Upregulated	Pancreatic cancer	32 in mt-tRNA^Ser^, mt-tRNA^Thr^	Promote	[44]
			mt tRNA	Upregulated	Pancreatic cancer	C32 in tRNA	Promote	[44]
METTL1	m^7^G	Writer	OXPHOS	Upregulated	Oral squamous cell carcinoma	mt-tRNA	Promote	[209]

## Data Availability

No datasets were generated or analysed during the current study.

## Abbreviations

ALKBH5: AlkB homolog 5
AMPK: AMP-activated protein kinase
APC: Adenomatous polyposis coli
BCAR4: Breast cancer anti-estrogen resistance 4
BPTF: Bromodomain PHD finger transcription factor
CALR: Calreticulin
COA1: Cytochrome c oxidase assembly factor 1
COX18: Cytochrome c oxidase assembly protein 18
COX6B2: Cytochrome c oxidase subunit 6B2
CTLA-4: Cytotoxic T-lymphocyte-associated antigen 4
DLGAP1-AS2: DLGAP1 antisense RNA 2
DRG8: Drosha/ DiGeorge syndrome critical region 8
EFTUD2: Elongation factor Tu GTP binding domain containing 2
ENO2: Enolase 2
ERK2: Extracellular signal-regulated kinase 2
ETC: Electron transport chain
FMR1: Fragile X messenger ribonucleoprotein 1
FOXP3: Forkhead box P3
FTO: Fat mass and obesity-associated protein
GPI: Glucose-6-phosphate isomerase
GLS: Glutaminase
GLUT: Glucose transporter
GSK3β: Glycogen synthase kinase 3 beta
HDGF: Hepatoma-derived growth factor
HIF-1α: Hypoxia-inducible factor 1 alpha
HK2: Hexokinase 2
IMS: Intermembrane space
KIAA1429: Key mediator of N6-methyladenosine modification methyltransferase complex subunit
LATS1: Large tumor suppressor kinase 1
LDH: Lactate dehydrogenase
MAPK: Mitogen-activated protein kinase
METTL3: Methyltransferase-like 3
NDUFA4: NADH: ubiquinone oxidoreductase subunit A4
NDUFS6: NADH: ubiquinone oxidoreductase subunit S6
NSCLC: Non-small cell lung cancer
NSUN2: NOP2/sun RNA methyltransferase family member 2
OXPHOS: Oxidative phosphorylation
PDH: Pyruvate dehydrogenase
PDK: Pyruvate dehydrogenase kinase
PFK: Phosphofructokinase
PFKL: Phosphofructokinase liver
PK: Pyruvate kinase
RBM15: RNA binding motif protein 15
ROS: Reactive oxygen species
RUNX1: Runt-related transcription factor 1
SOGA1: Suppressor of glucose by autophagy
TCA: Tricarboxylic acid
TCF/LEF: T cell factor/lymphoid enhancing factor
TEAD: Transcriptional enhanced associated domain
TFB1M: Transcription factor B1, mitochondrial
TME: Tumor microenvironment
UTR: Untranslated region
WWP2: WW domain-containing protein 2
YAP/TAZ: Yes-associated protein/transcriptional coactivator with PDZ-binding motif
YTHDF: YTH domain family protein
YTHDC1: YTH domain-containing protein 1
ZFP36: Zinc finger protein 36
